# A Reverse Taxonomic Approach to Assess Macrofaunal Distribution Patterns in Abyssal Pacific Polymetallic Nodule Fields

**DOI:** 10.1371/journal.pone.0117790

**Published:** 2015-02-11

**Authors:** Annika Janssen, Stefanie Kaiser, Karin Meißner, Nils Brenke, Lenaick Menot, Pedro Martínez Arbizu

**Affiliations:** 1 Senckenberg am Meer, Deutsches Zentrum für Marine Biodiversitätsforschung, Südstrand 44, 26382, Wilhelmshaven, Germany; 2 Senckenberg am Meer, Deutsches Zentrum für Marine Biodiversitätsforschung, Biozentrum Grindel, Martin-Luther-King Platz 3, 20146, Hamburg, Germany; 3 Institut Français de Recherche pour l´Exploitation de la Mer, Centre de Brest BP 70, 29280, Plouzané, France; University of Waikato (National Institute of Water and Atmospheric Research), NEW ZEALAND

## Abstract

Heightened interest in the exploitation of deep seafloor minerals is raising questions on the consequences for the resident fauna. Assessing species ranges and determination of processes underlying current species distributions are prerequisites to conservation planning and predicting faunal responses to changing environmental conditions. The abyssal central Pacific nodule belt, located between the Clarion and Clipperton Fracture Zones (CCZ), is an area prospected for mining of polymetallic nodules. We examined variations in genetic diversity and broad-scale connectivity of isopods and polychaetes across the CCZ. Faunal assemblages were studied from two mining claims (the eastern German and French license areas) located 1300 km apart and influenced by different productivity regimes. Using a reverse taxonomy approach based on DNA barcoding, we tested to what extent distance and large-scale changes in environmental parameters lead to differentiation in two macrofaunal taxa exhibiting different functions and life-history patterns. A fragment of the mitochondrial gene Cytochrome Oxidase Subunit 1 (COI) was analyzed. At a 97% threshold the molecular operational taxonomic units (MOTUs) corresponded well to morphological species. Molecular analyses indicated high local and regional diversity mostly because of large numbers of singletons in the samples. Consequently, variation in composition of genotypic clusters between sites was exceedingly large partly due to paucity of deep-sea sampling and faunal patchiness. A higher proportion of wide-ranging species in polychaetes was contrasted with mostly restricted distributions in isopods. Remarkably, several cryptic lineages appeared to be sympatric and occurred in taxa with putatively good dispersal abilities, whereas some brooding lineages revealed broad distributions across the CCZ. Geographic distance could explain variation in faunal connectivity between regions and sites to some extent, while assumed dispersal capabilities were not as important.

## Introduction

Steady increases in the demand of certain metals such as nickel, copper and cobalt during the last decade are raising the interest of exploring alternative mining sites like marine mineral deposits [1]. One type of deposit likely to be mined in the future is formed by polymetallic nodules, as they contain a relatively high proportion of these desirable metals [2,3]. Polymetallic nodule fields may cover large areas in the tropical abyss with the most important area for mining exploration being located in the central NE Pacific, between the Clarion and the Clipperton Fracture Zones (CCZ). The CCZ is characterized by gradual changes of environmental conditions (e.g. differences in surface-productivity, depth and sediment characteristics [4,5]) along an east-west as well as north-south axis, which lead to marked variation in nodule size and coverage, but also changes in faunal composition along these gradients [6]. The distribution, size and metal content of polymetallic nodules is determined by a variety of factors which include the degree of oxidation of the environment, the presence of nucleating agents and/or the nature and age of substrate, the proximity of sources of elements [7], sedimentation rates (which are largely influenced by the proximity to sources of sediment supply, overlying productivity and bottom current activity) and the influence of organisms [6–8]. Considering the different factors controlling the distribution of nodules, the rate of sedimentation and particulate organic carbon flux seem to be very important factors. Low sedimentation rates (0.3–0.5 cm/1000 y) correlate with high concentrations of nodules at the sediment surface, thus the highest nodule concentrations are usually found in red clay or siliceous areas [3].

Nodule mining will have an impact on the fauna in the mining area through removal of nodules, deposition of suspended sediment clouds during nodule extraction, as well as lifting and mobilization of metals. Thus, prior to mining-related exploitation, there is a need to obtain baseline data on faunal diversity, abundance and distributions to assess and predict the effects of mining on deep-sea organisms. As of May 2014, fifteen contractors are approved by the International Seabed Authority (ISA) [9] to explore nodule resources, with each license area covering up to 75,000 km^2^ [6]. The ISA requires every contractor to report on environmental status, current biodiversity of the area as well as population structure and standing stocks.

Several major research programs have investigated the biodiversity in nodule areas (e.g. Nodinaut [10–12]) and Kaplan [6]) to evaluate ecological baseline conditions and to provide recommendations on the protection of the nodule fauna prior to any potential commercial mining activities [13]. Small-scale impact experiments undertaken to date (such as DISCOL in the South-east Pacific Ocean [14,15], benthic impact experiments (BIEs) [16–18], the Japan deep-sea impact experiment (JET) [19] in the CCZ, and the Indian deep-sea environment experiment (INDEX) in the central Indian Ocean [20]) suggest that the environmental consequences of nodule mining will be severe and long-lasting [10,14,15]. Mining will furthermore affect large areas of the seafloor owing to direct mining disturbance (estimated scales of 300–600 km^2^ per year) and re-deposition from sediment plumes (over scales of 10–100 km from the mining site), which calls for a systematic conservation planning process and associated establishment of a marine protected area network and adjacent buffer zones across the CCZ [5]. Developing ecosystem management and systematic conservation plans for the deep sea however faces a number of key unknowns regarding the ecology of abyssal ecosystems, among which the evaluation of species’ ranges and levels of population connectivity are critical. Assessing species’ range size and population connectivity at abyssal depths is challenging because benthic communities are diverse, many species occur as singletons and most species are new to science (~90%, e.g. [21–24]). The taxonomic effort required to describe all these species would be tremendous; usually morpho-species or phenotypic clusters remain provisionally sorted, illustrated and numbered, which hinders timely morphological comparisons between highly diverse datasets and thus the assessment of regional diversity and biogeographic patterns [25].

Mining of manganese nodules is expected to begin within the next one or two decades [26]; thus a rapid characterization of the nodule fauna is required. This cannot be achieved in time by a traditional morphological approach alone, but by developing complementary tools and approaches to speed-up the identification process.

Here, we examined macrofaunal assemblages in two widely spaced mining claims (the eastern German and French license areas) in the CCZ, separated by 1300 km. These areas vary significantly in depth and surface productivity (oligo- vs. mesotrophic) which are likely to affect faunal diversity and distributional patterns. In our study, we investigated polychaetes and isopod crustaceans, since these taxa dominate abyssal macrofaunal abundance and species richness [27–31] while displaying different functional and reproductive strategies [32]. A reverse taxonomic approach was applied to estimate organismic diversity using genetic information [33]. This method allows for straight-forward allocation of individuals to genotypic clusters and thus facilitates comparison, overcoming the time-consuming morphological approach. First, distributional ranges of polychaete and isopod species as well as the similarity of molecular operational taxonomic units (MOTUs) between and within study sites were determined based on molecular methods. Subsequently the identities of shared MOTUs were analyzed using traditional morphological methods in order to test the value of reverse taxonomy for assessment of local and regional diversity as well as species ranges in the CCZ.

Compared to shallow-water systems, abyssal plains exhibit considerably lower rates of spatial variation in environmental parameters, leading to the perception of wider species distributions and greater genetic homogeneity with increasing depth [34,35]. Furthermore there are no major (continuous) topographic barriers across the CCZ. Thus, our null hypothesis (H_0_) is that species ranges are large; i.e. that dissimilarity of genotypic clusters (MOTUs) and genetic distance is not correlated with geographic distances and is generally low.

## Material and Methods

### Study area

This study was conducted in the high seas in areas beyond national jurisdiction (i.e. the Area in UNCLOS (United Nations Convention on the Law of the Sea) terminology), which are managed by the United Nations International Seabed Authority (ISA). Both Germany (through the Federal Institute for Geosciences and Natural Resources, Germany (BGR)) and France (through the Institut Français de Recherche pour l´Exploitation de la Mer, France (IFREMER)) have been granted exploration licenses in the CCZ. For this field study, all necessary permits were received from the BGR (Dr. C. Rühlemann) and IFREMER (Dr. L. Menot).

Benthic material was obtained during the complementary scientific cruises MANGAN (RV “Sonne”, SO205, Federal Ministry for Education and Research (BMBF)) in 2010 (March-May) and BioNod´12 (RV L’Atalante, BGR and IFREMER) in 2012 (March-May).

**Fig 1:**
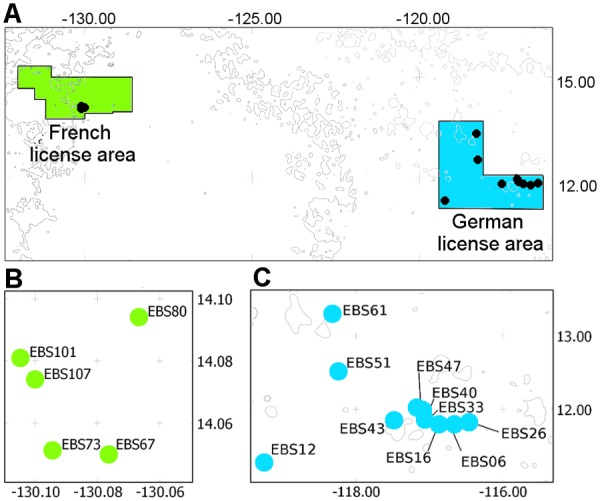
Study area. A Sampling locations in the German and French license areas; B Detailed map of sampling locations in French license area; C Detailed map of sampling locations in German license area.

The study areas are located between the Clarion and Clipperton Fracture Zone (CCZ, 6° N and 20°N, 120°W and 160°W) in the northeast equatorial Pacific Ocean at depths varying from 4358 m to 5055 m. The eastern German (centered on ≈12°N, 118°W) and the eastern French (centered on ≈14°N, 130°W) license areas are separated by 1300 km (Fig. 1A). Specific sample locations are given in Table 1.

**Table 1:** List of stations and successfully sequenced organisms.

license area	station	exped.	date	start lat (°N)	start long (°W)	depth (m)	Trawling distance (m)	no of polychaetes	no of isopods
German	EBS12	Mangan	28.04.2010	11.30111	-119.25	4403	2115	**65**	**0**
German	EBS26	Mangan	02.05.2010	11.830833	-116.447222	4221	2174	**80**	**0**
German	EBS40	Mangan	05.05.2010	11.938055	-117.052222	3954	463	**3**	**0**
German	EBS47	Mangan	07.05.2010	12.031944	-117.108333	4128	672	**51**	**0**
German	EBS61	Mangan	16.05.2010	13.304722	-118.309444	4280	2506	**40**	**0**
German	EBS06	BioNod12	02.04.2012	11.770345	-116.685561	4259	2271	**55**	**27**
German	EBS16	BioNod12	04.04.2012	11.794859	-116.885527	4135	2439	**19**	**2**
German	EBS33	BioNod12	07.04.2012	11.862434	-117.052893	4133	1917	**45**	**14**
German	EBS43	BioNod12	09.04.2012	11.803588	-117.534345	4358	2163	**47**	**33**
German	EBS51	BioNod12	11.04.2012	12.520562	-118.223339	4274	2307	**31**	**0**
French	EBS67	BioNod12	19.04.2012	14.051210	-130.076788	5021	3009	**2**	**15**
French	EBS73	BioNod12	20.04.2012	14.051245	-130.094259	5027	3459	**11**	**23**
French	EBS80	BioNod12	21.04.2012	14.093988	-130.066574	4986	3261	**18**	**10**
French	EBS101	BioNod12	25.04.2012	14.080841	-130.10455	5055	2937	**34**	**17**
French	EBS107	BioNod12	26.04.2012	14.073819	-130.099879	5023	3879	**55**	**9**
								**556**	**150**

### Study taxa

Polychaetes are the most common infaunal organisms; they show a wide range of functional strategies, from motile predators to tubiculous suspension feeders [32] and life-history strategies, from brooding with direct development to free spawning with planktotrophic larvae [36]. However, information on the biology and ecology of deep-sea species is sparse and often inferred from knowledge of shallow-water species.

Asellotan isopods (i.e. the majority of deep-sea isopods) are mainly detritivores and foraminiferivores [37,38]. They brood their young externally in a ventral brood pouch and follow either an infaunal or epifaunal lifestyle (i.e. living in or on the sediment). Data on reproduction patterns in deep-sea isopods are very limited; continuous recruitment throughout the year and several broods within a lifetime (iteroparity) has been reported [39,40], with each brood yielding up to 30 eggs [39,41].

### Data sampling and material processing

A Brenke-type epibenthic sledge (EBS) [42] was used to collect macrofaunal organisms from the eastern German (10 deployments, Fig. 1C) and eastern French (5 deployments, Fig. 1B) license areas following standard deployment procedures [42]. Shipboard, cod ends of the supra- and epi-net were sieved through a 500 μm- and 300 μm-mesh with cold (+10°C) sea water and immediately transferred to pre-cooled (-20°C) 96% EtOH. These samples were stored in -20°C for at least 48 h for later DNA extractions. After 24 hours, the ethanol was decanted and replaced with new 96% EtOH to guarantee preservation of high-quality DNA. Subsequently, the samples were kept at -20°C until further sample processing.

The samples collected by the EBS were sorted into separate taxa onboard and in the laboratories of Senckenberg am Meer, DZMB, Wilhelmshaven and Hamburg, Germany. A total of 1380 polychaetes and 520 isopod individuals were photographed using Leica binocular microscopes, DNA was extracted and Cytochrome-c-oxidase Subunit 1 (COI) was amplified.

Genotypic clusters or MOTUs represented by more than one specimen, were morphologically discriminated to lowest taxonomic level possible, but ultimately leading to phenotypic clusters (i.e. morpho-species categories) [43].

### Molecular analyses

To receive a sufficient amount of tissue, but also allow further morphological analyses, we used a semi-destructive approach; one to three legs (unilateral, pereopods 2–4 only) were dissected from each isopod specimen, while one to three parapodia or smaller tissue samples were taken from the middle part of each polychaete, depending on the size of the individual. According to the tissue samples, the organisms were designated collection numbers (S1 Table, S2 Table). The voucher specimens are stored at 4–8°C, tissue and DNA-samples are stored at -20°C in the laboratories of Senckenberg am Meer, Wilhelmshaven and Hamburg, Germany.

### Extraction

Chelex 100 BioRad was used to extract DNA from tissue samples [44]. The DNA was extracted according to the following protocol: 1. tissue was dissected from the organism and then washed in distilled water several times; 2. tissue was transferred into 30 μl of Chelex in 0.2 ml PCR tubes; 3. this was followed by incubation in a thermocycler: for 60 min at 56°C, followed by boiling for 10 min at 99°C; 4. the sample was centrifuged for 30 s at 6000 rpm; 5. the supernatant was used as template for amplification.

### Polymerase Chain Reaction (PCR)

A fragment of the mitochondrial COI was amplified using universal primers forward: LCO1490 5′-ggtcaacaaatcataaagatattgg-3′; reverse: HC02198: 5′-taaacttcagggtgaccaaaaaatca-3′ [45]. The PCR was conducted according to the following protocol: 22 μl sterile distilled water, 0.5 μl forward primer, 0.5 μl reverse primer und 2 μl DNA-template were filled on ice in 0.2 μl PCR tubes with illustra^TM^PuReTaq^TM^Ready-to-Go^TM^PCRbeads; the sample was centrifuged for 30 s at max 6000 rpm; the incubation in the thermocycler was proceeded by the following cycles step 1: heating to 95°C for 5 min (denaturation); step 2: repeating of step 1 for 30 s (denaturation); step 3: cooling down to 42°C for 1 min (annealing); step 4: heating again to 72°C for 1 min (elongation); step 5: repeating step 2–4 for 40 cycles; step 6: continuing at 72°C for further 7 min (elongation) and step 7: cooling down to 4°C (deletion of enzymes).

### Sequencing

PCR-products, which produced light bands after electrophoresis on 1% agarose gel, were sent to the MacroGen Europe Laboratory in Amsterdam, Netherlands for sequencing using the same set of primers as used for the PCR.

### Treatment of sequences

Sequences were processed and aligned with Geneious Pro 6.0.5 2005–2012 Biomatters Ltd. (MUSCLE alignment) (available from http://www.geneious.com/). The datasets were translated into amino acid alignment and checked for stop codons to avoid pseudogenes. The phylogenetic trees (Neighbor Joining, p-distance) were created with the program MEGA 5.2.2. [46] and edited with the online-tool Interactive tree of life (iTOL) [47]. The online-tool CD-HIT-Suite [48] was used for quickly identifying MOTUs using pairwise alignment with a defined similarity threshold. CD-HIT first sorts sequences in decreasing length order. The longest sequence becomes the representative of the first cluster. Then, each sequence is compared pairwise to the first one. Sequences are assigned to a cluster based on a pre-defined threshold. If sequence similarity exceeds this threshold, it is assigned to this cluster. Otherwise, a new cluster is defined using this sequence as a reference. The pre-defined threshold for this analysis was 0.97 [48,49] or 97% similarity, which also corresponds to the universal DNA-barcoding threshold proposed by Hebert and co-workers [50]. The minimum length coverage was set to 400bp.

Average sequence divergence between MOTUs was estimated using the Kimura two-parameters (K2P) model of base substitution [51]. For further comparisons with other studies, uncorrected p-distances were calculated. Both were computed using MEGA 5.2.2.

All sequences obtained in this study have been deposited in GenBank under accession numbers KJ736018–KJ736723 (S1 Table, S2 Table).

### Genetic similarity between sites

We computed the unweighted UniFrac metrics using the freeware Fast UniFrac [52] in order to test for genetic similarity between samples. Based upon a phylogenetic tree, the UniFrac metric measures the difference between two or more samples in terms of the overall branch length that is unique to each sample. If the division of samples occurs at the basal-most node of the tree, so that all of the branch length is uniquely allocated to the respective samples, the UniFrac metric will result in the maximum distance possible (1.0). At the other extreme, if all terminals are shared between all samples, (i.e. all nodes have descendants in each of the samples), the UniFrac distance will result in the minimum distance possible (0.0) [52]. UniFrac distance between samples was visualized by non-metric Multidimensional Scaling (nMDS) [53]. In order to test for significant differences of pre-defined groupings in relation to region, a one-way analysis of similarity (ANOSIM) [54] was conducted in Primer 6.0 [53].

Finally, we tested the correlation between input distance matrices of samples using the non-parametric Mantel test (through 5000 permutations) [55]. Correlations have been tested pairwise a) between the genetic distances (of individual sequences) and the geographic distances, b) between the similarity of MOTUs and geographic distance as well as c) between genetic distance and similarity of MOTUs. For computing the distance matrices, the Cosine Index was used for similarity of MOTUs, Euclidean distance for geographic distance, and UniFrac metric for genetic distance. The test statistic is the Pearson product-moment correlation coefficient r, which falls in the range of-1 to +1. Close to-1 indicates strong negative correlation and +1 indicates strong positive correlation. An r value of 0 indicates no correlation. All Mantel tests were performed by the free software PASSaGE [56].

### Similarity analysis

The similarity analysis of MOTUs between sites was computed using the cosine similarity [57]. Each sample is represented by an ordered vector (composed by the abundance of the MOTUs). As a qualitative measure, the similarity between samples is represented algebraically by the dot product of their sample vectors, which equals the cosine of the angle between the vectors representing the position of the samples in a multidimensional species-space. The value of the cosine similarity in this study is always positive as no negative MOTU presence can be recorded. It ranges between 0 for orthogonal vectors (sharing no MOTUs) and 1 for vectors with same orientation (sharing all MOTUs). The cosine similarity, being a measure of the collinearity (direction) of the vectors, rather than a measure of differences in the magnitude of the vectors, does not downgrade the importance of low-abundance species (MOTUs) and considers only shared attributes. This is appropriate to our study, because the majority of species are represented by singletons. Furthermore the EBS, as a non-quantitative sampling device, does not allow for quantitative analyses [42]. The similarity matrix was visualized with nMDS and significant differences were tested by ANOSIM [54] using the free software PAST [58].

### Diversity analyses

For estimations of genetic diversity of pooled samples (i.e. German and French samples respectively) we calculated species richness (S) (as in number of genotypic clusters/MOTUs). Due to the non-quantitative sampling gear used, the data set does not reflect original community structure. EBS sample diversity does not reflect alpha-diversity in terms of species richness nor species density, but reflects rather the local species pool over trawled distance. A valid diversity comparison is the estimated species richness at each license area (regional diversity), using richness estimators [59]. We performed one abundance estimator Chao 1 [60] and two incidence estimators, Chao 2 [61] and Jackknife 1 [62], to estimate a rough number of expected species in the region. The extrapolative methods were performed in PRIMER 6.0 [53]. All graphical illustrations were revised with GIMP.

## Results

### Molecular analyses

From the 1900 specimens analyzed, COI amplification and sequencing was successful for 556 polychaete and 150 isopod specimens, i.e. 44% and 31% of the total number of polychaetes and isopods selected for genetic analyses, respectively (Table 1). Of these, 436 polychaete and 76 isopod sequences were obtained from the German license area, whereas 120 polychaete and 74 isopod sequences could be retrieved from the French license area (Table 1). The majority of sequences has a sequence length of approximately 650 bp. Searching the reference sequence of every MOTU against GenBank [63] using the BLAST algorithm, resulted in very low percentage matching success, probably reflecting generally high rates of novelty of abyssal species as well as scarcity of deep-sea data in GenBank (cf. [64,65]). The sequence of polychaete voucher EBS61o-Po88 (reference sequence of cluster 2) corresponded to a known species of Goniadidae, i.e. *Bathyglycinde profunda* (Hartman & Fauchald, 1971) [66] with a sequence similarity of 99.2% (GenBank Accession No. GQ426633). This species has been previously recorded from the continental slope and abyss of the Atlantic Ocean (i.e. Bermuda, Suriname, Brazil) [67] and the CCZ [6].

### Polychaeta

Analyzed polychaete specimens belonged to 21 supraspecific taxa: Acrocirridae, Opheliidae, Nereididae, Goniadidae, Ampharetidae, Hesionidae, Spionidae, Magelonidae, Paralacydonidae, Pholoidae, Poecilochaetidae, Polynoidae, Paraonidae, Sphaerodoridae, Scalibregmatidae, Cirratulidae, Sigalionidae, Sabellidae, Phyllodocidae, Nephtyidae and Lacydoniidae (S3 Table).

Most abundant MOTUs represented in both the French and German license areas were morphologically assigned to *Paralacydonia* cf. *weberi* (Horst, 1923) [68] (with relative abundance (RAD) of 14% (excluding singletons)) and *Bathyglycinde profunda* (9% RAD). The most abundant taxa shared between the French and German license areas were Opheliidae (19% RAD), Spionidae (17% RAD) and Paralacydonidae (14% RAD).

**Fig 2:**
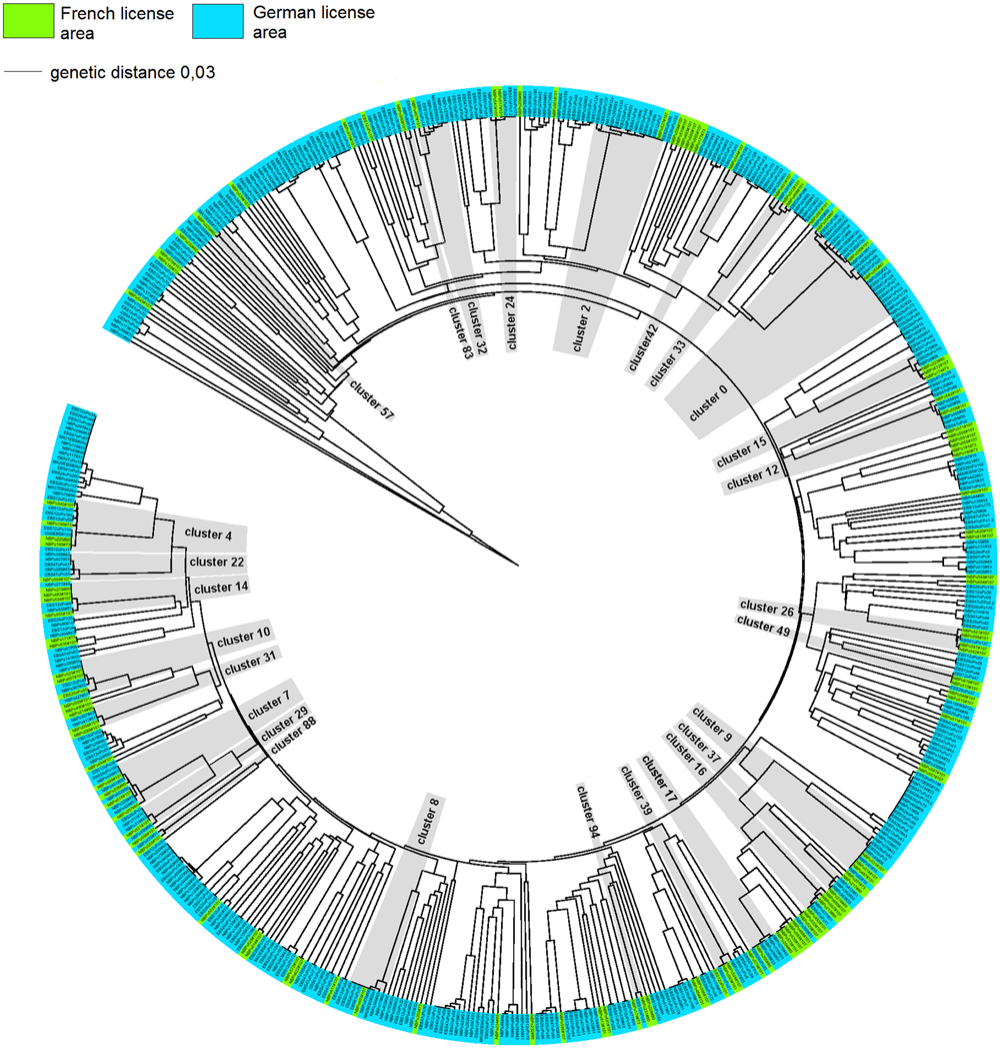
Molecular Neighbor Joining tree (p-distance) of COI polychaete sequences, including marked CD-Hit cluster (MOTUs). Highlighted clusters are those sharing MOTUs only across license areas at a similarity threshold of 97%.

Using CD-HIT, the 556 COI sequences clustered into 233 MOTUs at a similarity level of ≥ 97%. Of the 233 MOTUs, a total of 95 MOTUs (40%) were represented by more than one sequence, while 138 sequences were singletons (i.e. 60% of MOTUs, but 25% of total sequences were recorded only once) (Fig. 2). Out of 95 MOTUs found more than once, 27 MOTUs (~28%) were found in both the French and German license area, 56 MOTUs (~59%) were exclusively found in the German license area, and 12 MOTUs (~13%) occurred only in the French license area. Including singletons, 158 MOTUs were present exclusively in the German license area compared to 48 in the French license area.

The aim of the taxonomic species determination was to provide quality control of the CD-Hit (genotypic) clustering by comparing the genotypic clusters with phenotypic clusters. Species identification was hampered by the fact that most species occurring in the investigation area are either poorly known or new to science. Due to their poor condition some specimens could not be reliably identified and were referred to as ‘species indet’. Furthermore, various characters important for species identification of polychaetes are not preserved in 96% ethanol (which was necessary for molecular analyses), but require formalin-based fixation. Thus in most cases identification to genus level was undertaken.

**Fig 3:**
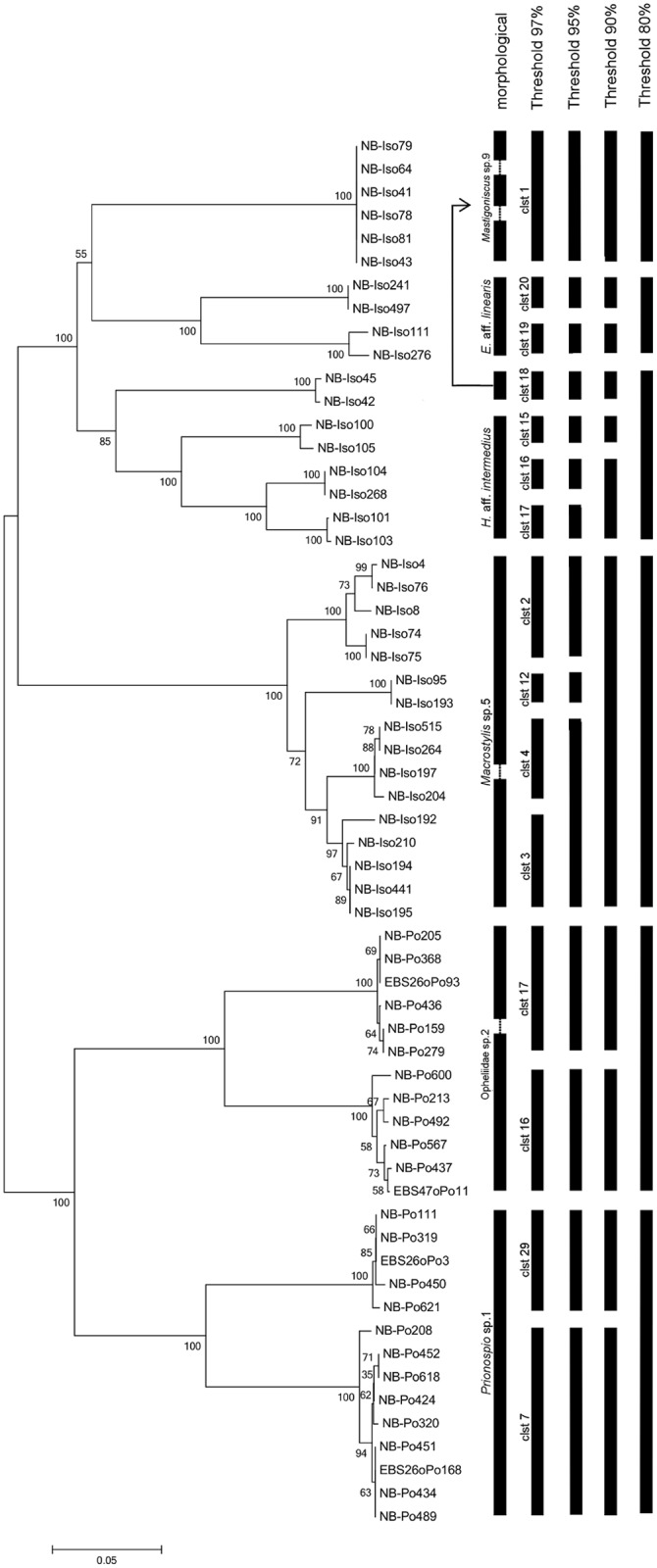
Comparison between morphological determination and MOTUs with different thresholds. Neighbor Joining tree (p-distance) of clusters which do not coincide for morphological and molecular determinations at 97% sequence similarity.

The morphological analyses supported the CD-Hit clustering in the majority of cases. The 95 MOTUs, for which more than one individual was found, could be matched with 95 morpho-species (phenotypic clusters). In two MOTUs (17 and 83), which display a sequence similarity of 99%, a single sequence each did not correspond to the morphological examination (S3 Table). Although the sequence similarity was very high, these individuals were morphologically allocated to Opheliidae and Spionidae (5 specimens versus 1 specimen in cluster 17) and to *Laonice* and *Prionospio* (one specimens each, both belonging to Spionidae (cluster 83)), respectively. On the other hand, specimens identified as morpho-species Opheliidae sp.2 and *Prionospio* sp.1 based on the available material were both assigned to two different clusters by CD-Hit clustering (S3 Table). A comparison of morphological and genetic identifications for Opheliidae sp.2 and *Prionospio* sp.1 revealed that analogous results could only be obtained at very low threshold values (≤ 80%) (Fig. 3). The mean sequence distance comparisons (K2P) within morpho-species Opheliidae sp.2 and *Prionospio* sp.1 were 9.4% and 12.5% respectively. Interspecific comparisons between both morpho-species (within polychaete and isopod taxa, respectively) were much higher and ranged from 33.3–37.4% (Table 2). Uncorrected p-distances were almost similar to calculated K2P distances (results not shown).

**Table 2:** Mean sequence distances comparisons based on Kimura-2-parameters (in %) between analyzed taxa.

	Opheliidae sp.2	*Prionospio* sp.1	*Mastigoniscus* sp.9	*E*. aff. *linearis*	*H*. aff. *intermedius*	*Macrostylis* sp.5
Opheliidae sp.2	9.4	—	—	—	—	—
*Prionospio* sp.1	35.5	12.5	—	—	—	—
*Mastigoniscus* sp. 9	—	—	11.6	—	—	—
*Eurycope*.aff. *linearis*	—	—	30	11.1	—	—
*Haploniscus* aff. *intermedius*	—	—	27.6	29.9	9.2	—
*Macrostylis* sp.5	—	—	40.5	41.5	38.6	9.6

**Fig 4:**
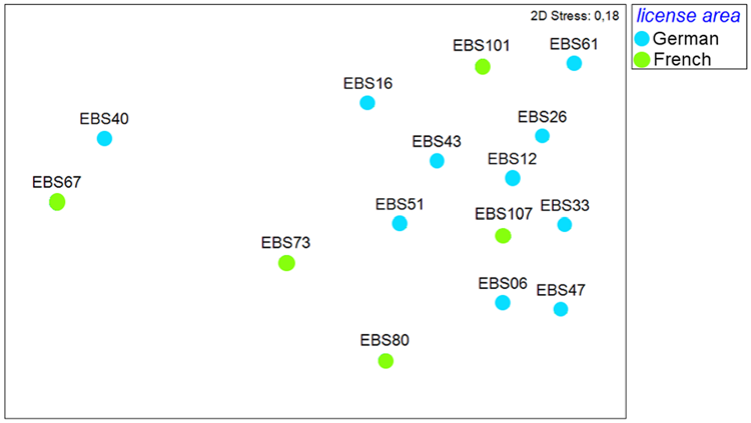
Non-metric multidimensional scaling (nMDS) plot for similarity of samples of polychaete sequences, based on UniFrac metric.

The nMDS of unweighted UniFrac metric (Fig. 4) displayed no evident grouping of the French and German stations. The pairwise one-way analysis of similarities (ANOSIM) test showed that the genetic distance of polychaete assemblages between the German and the French license areas did not differ significantly from chance (Global R = 0.13, p = 0.15, number of permutations = 999, Table 3), that is the within-group variability was as high as the between-groups variability.

However, a low but significant positive correlation (r = 0.32, p < 0.01) in terms of genetic and geographical distances between all stations has been detected applying the Mantel test (Table 4). This indicates that an increase in geographic distance predicts a slight increase in the genetic distance.

**Table 3:** Pairwise one-way analysis of similarity (ANOSIM) for German and French license area, computed with UniFrac metric.

**Polychaeta**	German license area
	Global R
French license area	0.13
**Isopoda**	German license area
	Global R
French license area	0.38

### Isopoda

**Fig 5:**
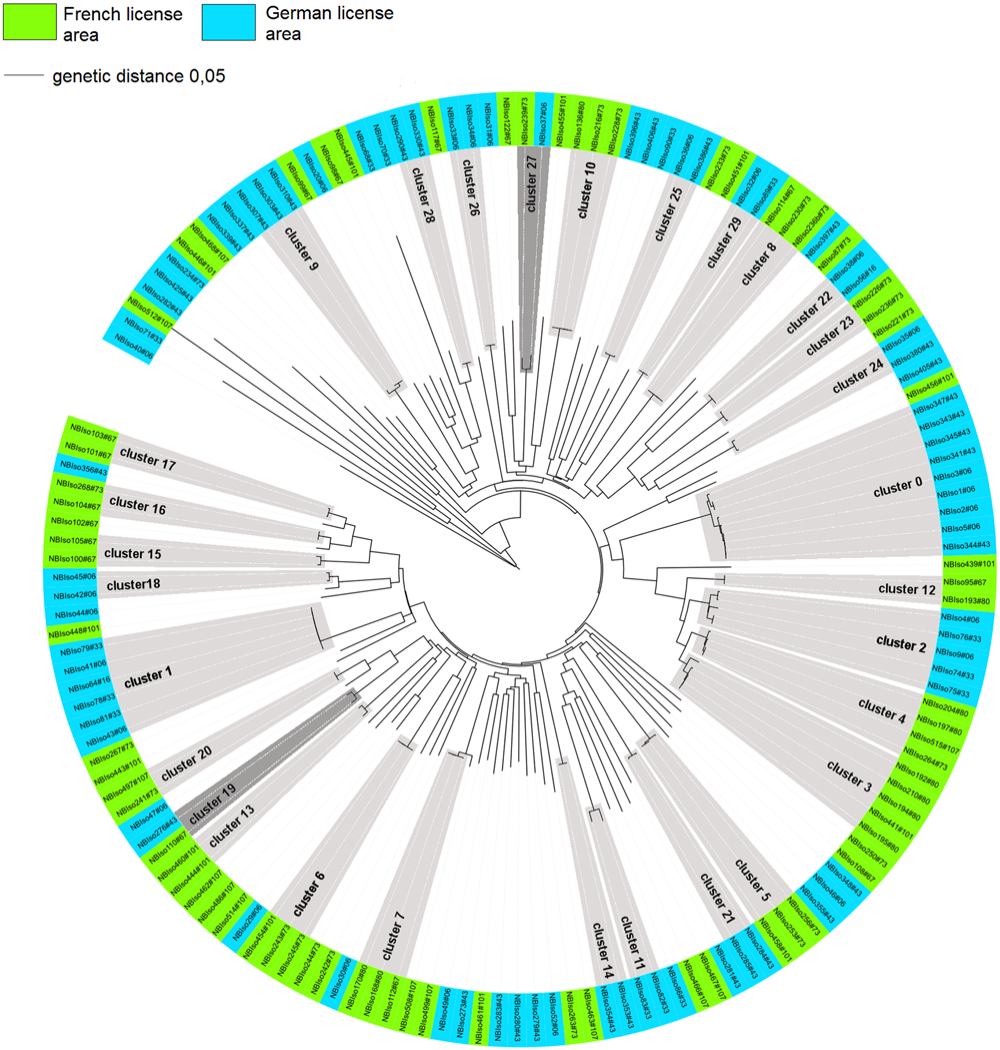
Molecular Neighbor Joining tree (p-distance) of COI isopod sequences, including marked CD-Hit cluster (MOTUs). All MOTUs at a similarity threshold of 97% except singletons are highlighted; light grey: only shared within-license area; dark grey: shared across license areas.

In Isopoda, analyses indicated seven supraspecific taxa: Desmosomatidae, Macrostylidae, Munnopsidae, Nannoniscidae, Haploniscidae, Haplomunnidae and Dendrotionidae (S4 Table). The most abundant morpho-species were assigned to a new species of Macrostylidae (*Macrostylis* sp.5; 15% RAD (excluding singletons)). Most abundant taxa, for which sequences could be obtained and which were recorded in both the French and German license areas, were Macrostylidae (29% RAD) and Desmosomatidae (26% RAD). In total we identified 95 MOTUs (at ≥ 97% similarity). Of those, 30 MOTUs (~ 30%) were represented by more than one sequence and 65 MOTUs were singletons (~70% of MOTUs, ~50% of total sequences). Out of the 30 MOTUs that were represented by more than one individual, only two MOTUs (7%) were present in both the German and French license areas (i.e., *Eurycope* aff. *linearis* and *Prochelator* sp. 1, S4 Table). This is in contrast to morphological analyses, which recorded three species occurring in both the French and German claims. Fourteen MOTUs (47.5%) were found exclusively in the French and 14 MOTUs (47.5%) were restricted to the German license area (Fig. 5). Including singletons, 45 MOTUs were found exclusively in the German and 48 in the French license area.

All clusters with more than one specimen were morphologically analyzed (S4 Table). The morphological determination supported CD-Hit clustering in the majority of cases, but revealed larger discrepancies between genetic and morphological discriminations than observed for polychaetes. The 30 MOTUs that were recorded more than once could be annotated to 26 morpho-species. The genotypic clusters 1, 4, 7, and 23 included at least one individual respectively that displayed distinctive morphological characters, although sequence identity was 99–100%. In each case, however, generic identification was consistent. In the case of cluster 23, the mismatch between genetic and morphological discrimination may be due to damage of the voucher specimen, so that the individual could not be reliably identified. Furthermore, CD-Hit clustering separated several morphologically similar specimens into different genotypic clusters (i.e. *Macrostylis* sp.5, *Haploniscus* aff. *intermedius* Birstein, 1971, *Eurycope* aff. *linearis* Birstein, 1963, *Mastigoniscus* sp.9, S4 Table). By lowering the threshold to 90%, morphological identification was in agreement with genotypic clusters *in Macrostylis* sp.5. In *Haploniscus* aff. *intermedius*, *Eurycope* aff. *linearis* and *Mastigoniscus* sp.9 the threshold had to be reduced to 80% to receive concordant results (Fig. 3). Cluster 18, which was morphologically related to *Mastigoniscus* sp.9, could not be assigned to cluster 1 at any threshold, but rather to cluster 15, 16 and 17, which were morphologically determined as *Haploniscus* aff. *intermedius*. Mean sequence distance (K2P) comparisons within morpho-species *H*. aff. *intermedius*, *E*. aff. *linearis*, *Mastigoniscus* sp.9 *and Macrostylis* sp. 5 were 9.2%, 11.1%, 11.6% and 9.6% respectively. Interspecific comparisons between these morpho-species were much higher and ranged from 27.6% to 41.5% (Table 2). Like in polychaetes, the K2P distances were in accordance with uncorrected p-distances, and thus are not shown.

**Fig 6:**
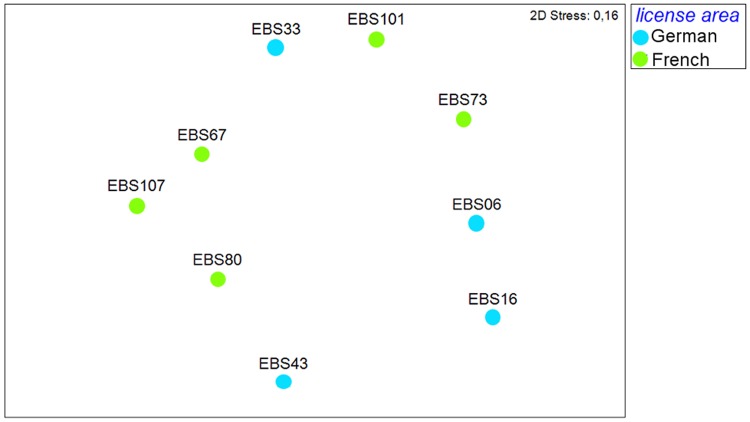
Non-metric multidimensional scaling (nMDS) plot for similarity of samples of isopod sequences, based on UniFrac metric.

The nMDS unweighted UniFrac metric (Fig. 6) showed a slight grouping of the French and German isopod assemblages. The ANOSIM test showed that the genetic isopod assemblages of the German and the French areas differ significantly (one-way ANOSIM, Global R = 0.38, p = 0.016, number of permutations = 999), but the R value is low.

The Mantel test revealed a significant positive correlation (r = 0.56, p < 0.01) of genetic and geographical distances between all stations and thus confirms the previous findings. This positive correlation was found to be stronger for isopods than for the polychaetes (Table 4).

**Table 4:** Mantel-Test.

**r-values**	SimMatCosPoly	SimMatCosIso	UniFracDistPoly	UniFracDistIso
geoDistEucl	-0.28243	-0.24047	0.3231	0.562
SimMatCosPoly			-0.55958	
SimMatCosIso				-0.45390
**p-values**				
geoDistEucl	0.00703	0.04913	0.00212	0.00025
SimMatCosPoly			0.00001	
SimMatCosIso				0.01542

It was tested the correlation between genetic distance (UniFracDist) and geographic distances (geoDistEucl), between MOTU similarity (SimMatCos) and geographic distance (geoDistEucl) as well as between genetic distance (UniFracDist) and MOTU similarity (SimMatCos) for both polychaete and isopod organisms.

### Similarity analyses

**Fig 7:**
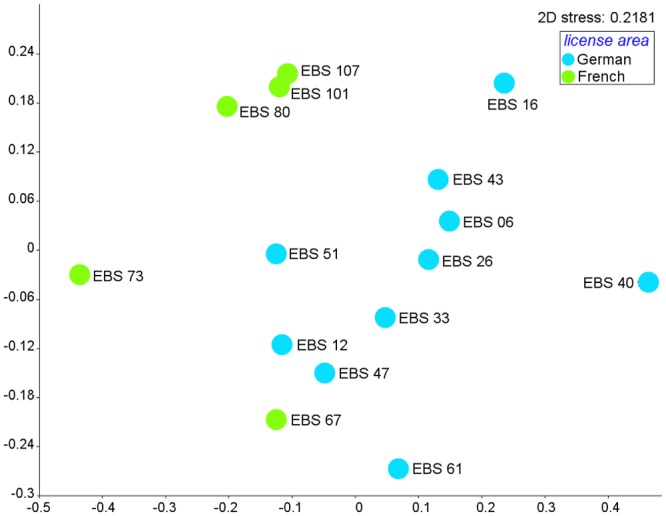
Non-metric multidimensional scaling (nMDS) plot for similarity of samples of polychaete MOTUs, based on Cosine Similarity.

The nMDS plot of cosine similarity for polychaete MOTUs (Fig. 7) illustrates that samples were slightly subdivided into two groups based on the respective license areas (French and German). The stress value, however, was high (0.21) emphasizing that the results are not well represented in a two-dimensional way. ANOSIM analyses revealed significant differences between polychaete MOTUs of the German and French license area (one-way ANOSIM, Global R: 0.3367; p < 0.05) (Table 5). The sample statistic was very low, though, probably reflecting great variability in similarity of MOTUs within both the German and the French claims.

**Table 5:** Pairwise one-way analysis of similarity (ANOSIM) for German and French license areas, computed with Cosine Similarity.

**Polychaeta**	German license area
	Global R
French license area	0.3367
**Isopoda**	German license area
	Global R
French license area	0.244

The Mantel test calculated a slightly negative (r = -0.28) but significant (p < 0.01) correlation between the cosine similarity and geographical distance, indicating that similarity of MOTUs between samples decreased with increasing geographical distance (Table 4). Furthermore the Mantel test confirmed also a relative strong negative and significant correlation (r = -0.55, p < 0.01) between the genetic distance and cosine similarity, which indicates increasing genetic distance with decreasing similarity of MOTUs (Table 4).

**Fig 8:**
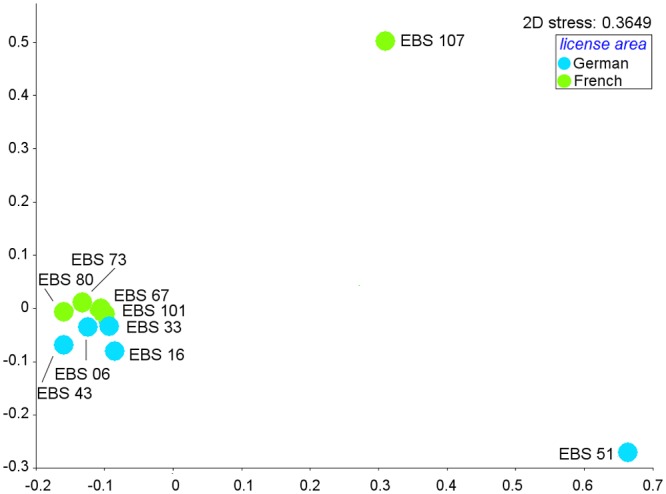
Non-metric multidimensional scaling (nMDS) plot for similarity of samples of isopod MOTUs, based on Cosine Similarity.

Both the nMDS plot of cosine similarity for isopods (Fig. 8) and ANOSIM (one-way-ANOSIM, Global R = 0.244, p < 0.01, numbers of permutations = 999, Table 5) showed a significant grouping of stations in the French and German license areas respectively, even though two stations (i.e. one from each area) were widely dispersed due to very low similarity of isopod assemblages to any other station (Fig. 8). Furthermore the 2-D stress value is high and thus differences between stations and areas are not well illustrated in the nMDS.

The Mantel test showed a significant and slightly negative correlation (r = -0.24, p < 0.05) between cosine similarity and geographical distance for isopods (Table 4). The Mantel test confirmed also a relatively strong negative and weakly significant correlation (r = -0.45, p < 0.05) between the genetic distance and cosine similarity, which suggests that the similarity of MOTUs become more dissimilar with increasing distance (Table 4).

### Diversity analyses

The expected species number in the two studied areas has been estimated with one abundance estimator: Chao1 and two incidence estimators: Chao2 and Jackknife 1. The estimated percentage of species recovered at German license area lies between 43.9% (Chao2) and 62.1% (Jackknife 1). For the French license area, the estimated recovered percentage is conspicuously lower and lies between 28.9% (Chao2) and 61% (Jackknife 1) (Table 6). Chao1 and Chao2 indices indicate that the number of collected species accounts for less than half of the species expected in the sampled areas.

**Table 6:** Expected species numbers (± standard deviation) as calculated by three different extrapolative estimation methods (Chao1, Chao2, Jackknife), plus the proportion of those collected.

	German license area	French license area
*S_obs_*	233	122
N	511	195
Chao1	525 ± 71	327 ± 67
%registration	44.4%	37.3%
Chao2	530 ± 68	422 ± 98
%registration	43.9%	28.9%
Jackknife 1	375	200
%registration	62.1%	61%

## Discussion

### Reverse taxonomy—merits and pitfalls

In the marine realm, molecular genetic tools are increasingly used alongside classical taxonomy for species identification and delimitation, and thus for estimating levels of biological diversity and distributional ranges [69]. In the deep sea, with its huge yet largely unknown biodiversity, DNA taxonomy seems to be a promising tool to accelerate biodiversity assessment [25,33,70–73]. Species are the fundamental units for biodiversity research [43] and for unambiguous use of the term species, species concepts need to be referred to. The knowledge about distribution patterns of deep-sea species is scarce in most cases due to the paucity of data. Consequently, necessary evidence or even assumptions required to match the species criteria imposed by the many species concepts under discussion (e.g. [74]) are unavailable in most cases. Nevertheless, provisional species delimitation is required for diversity studies and thus operational criteria were adopted in this study. Both the genotypic and phenotypic cluster definitions [74,75] provide such operational criteria.

Here, a reverse taxonomy approach was applied to explore levels of regional diversity and species ranges of two dominant macrofaunal taxa inhabiting the abyssal Pacific nodule province. This method has been previously applied in both ecological and taxonomic surveys of assemblages and taxa, which are difficult to discriminate based on morphological features only (e.g. [33,76,77]). The advantage of MOTU surveys is that they can be automated and (arguably) do not require any taxonomic specialist knowledge [33]; that is MOTUs are initially defined by genetic means (as genotypic clusters), while morphological identities and thus ecological functions, remain unknown—at least at first [33]. Sequences could be then compared to a reference data base (e.g. GenBank), and named according to the existing phylogenetic framework [73]. However, DNA barcodes can only assign MOTUs to sequences of known species listed in a data base, or distinguish different MOTUs, but do not relate them to a species name if the identity is unknown. As the vast majority of species in our study appears to be new to science and sequence data in GenBank are scarce for most deep-sea taxa (cf. [64]), a close integration of morphological and genetic methods is crucial for accurate species delineation as a baseline, at least until a data base for the CCZ can be established to allow a straight-forward DNA barcoding approach [50].

Despite relatively low sequencing success, this analysis represents probably one of the most comprehensive studies investigating long-range distribution of species in polymetallic nodule fields. The low number of sequence data is an obstacle for receiving statistically powerful results especially with regard to estimating inter- and intraspecific variability. Low numbers also impede accurate delineation of species based on morphological characters—again stressing a general problem in examining deep-sea connectivity. More samples and/or more unlinked (mitochondrial and nuclear) markers may help to obtain more robust results in the future.

Furthermore, comparison of morphological and molecular data is often problematic, as different fixatives (formalin vs. ethanol) are required for optimal preservation. The mere fact that many characters important for species identifications are not preserved in ethanol (especially in polychaetes) leads to the confinement that voucher specimens (fixed in ethanol) cannot necessarily be related to morpho-species identifications in other studies using formalin-preserved samples.

In our study, MOTUs mostly corresponded to morphologically identified species at a sequence similarity of 97%, which is equivalent to the “universal” threshold for species delineation proposed by Hebert (2003) [50], highlighting the high power of COI for species level discrimination. Even where morphological and genetic identifications were not in congruence, MOTUs could be correctly annotated to supraspecific taxa (i.e. genera; S3 Table, S4 Table). Two exceptions had to be made for polychaete clusters 17 and 83, where within-cluster specimens were partially assigned to different supraspecific taxa (i.e. ‘families’, genera, S3 Table). This mismatch could not be resolved. However these specimens can be neglected from quantitative point of view, as they just represent a small proportion of the molecular data set (two out of 706 specimens).

In some cases, morpho-species were distributed across different genotypic clusters as inferred by K2P distances ranging from 9.2% to 12.5% mean distance between clusters; these may indicate genetically divergent species in isopods and polychaetes that could be interpreted as lineages of young age and hence still morphologically undifferentiated. In asellotan isopods, uncorrected p-distances between COI sequences typically show intraspecific variation ranging from 0 to 1.8% and from 9 to 20% between species [64,78], while intergeneric distances varied from 25 to 28% [64]. For polychaetes, Carr et al. [79] reported mean values for intraspecific (K2P) divergence of 0.38%, whereas mean interspecific divergence was 16.5%. For Spionidae interspecific genetic distances at the COI locus ranged from 11.7% to 22.5% (uncorrected distance) [80–82]. Therefore, in comparison to mean distances between genotypic clusters (of same morpho-species) in this study, they would be very high for intraspecific and quite low for interspecific variance. However, these patterns of intra- and interspecific divergence observed in this and previous mentioned studies [64,78–82] are likely to be strongly biased by the scarcity of data available and do not show the full range of intra- and interspecific variation.

Nevertheless, the mitochondrial COI was proposed as universal barcoding marker [83] and since then has been highly attractive for molecular species discrimination and identification [84,85], due to generally high substitution rates, almost exclusively maternal inheritance and the lack of recombination [50,85–87]. Other genes such as ribosomal 16S, 18S and SSU rDNA (i.e. [88–90]) and mitochondrial Cyt b (i.e. [91,92]) have been also used as standard or as complementary DNA barcoding markers. The issue of arbitrary thresholds used to define MOTUs is a primary concern for molecular taxonomy, when intraspecific variation is high. Other approaches, i.e. general mixed Yule coalescent (GMYC) model or automatic barcode gap discovery (ABGD) are inappropriate to our data set, as it contains both a high number of MOTUs [85] and a low number of sequences per MOTU [93].

Considering the scenario of fifteen different nodule exploration contractors assessing the mostly undescribed biodiversity of benthic communities in their own license area within the CCZ, taxonomic standardization and intercalibration is a challenge but also a chance for joint efforts and unparalleled collections. The use of molecular barcodes, which can be exchanged between contractors or published in online repositories (GenBank, BOLD) is promising to be of tremendous help and thus should become a standard procedure. For now, using a combination of morphological and genetic approaches should provide the most robust estimates on biodiversity and species ranges.

### Factors determining species ranges across the CCZ

How species are distributed across large geographic ranges is poorly studied in the abyss, but there is some evidence for broad-scale distributions in a number of taxa [94,95]. Biogeographic patterns and species ranges seem to vary substantially for instance with body size [96–98]. For megafaunal organisms it is known that many species are widely distributed [94,99,100], whereas the distribution of macro- and meiofaunal species had been assumed to be much more restricted—potentially due the paucity of deep-sea sampling [94]. In recent years, molecular investigations on a number of macro- and meiofaunal taxa have revealed contrasting findings; some macro- and meiofaunal species seem to have wider distributions than previously thought [22,101–106], while others represent species complexes and thus ranges have become much more restricted [107,108]).

Based on both morphological and genetic markers, we examined levels of faunal connectivity in polychaetes and isopods, which both may be able to disperse over a long distance. Our null hypothesis of genetic homogeneity across large geographic distances was not confirmed. In contrast, we found significant differentiation in similarity of MOTUs between regions both in isopods and polychaetes (Fig. 7, Fig. 8). Nevertheless, our results support that at least some species (27 polychaete and two isopod species) have ranges extending from the eastern to the central part of the CCZ, which may be partly due to the lack of major geological barriers.

A variety of ecological and evolutionary explanations have been linked to variation in geographic ranges among species (such as evolutionary history, environmental variability, (micro-) habitat availability, niche breadth, colonization and extinction dynamics and population abundance [97,109,110]). High dispersal ability has been suggested to be a prime determinant of wide spatial distributions (e.g. [97]). The duration of pelagic larval stages has been often used as a proxy for dispersal potential with the latter decreasing from planktotrophy (feeding larvae), lecithotrophy (non-feeding larvae) to brooding development [111]. It seems though that larval dispersal does not always translate into a wide geographic distribution [112], while brooding does not necessarily imply a restricted range size (e.g. [64,113]).

The broad-scale distribution that we found in two isopod lineages (*Eurycope* aff. *linearis* and *Prochelator* sp.1) seems to be remarkable for brooders. In previous studies, molecular data of presumably widely distributed deep-sea isopod species revealed species complexes or cryptic species (e.g. [114,115]). However, there is also evidence for long-distance dispersal within brooding isopods. For example, some isopod species seem to be able to maintain gene flow between populations over large geographic distances (hundreds to thousands of kilometers), across major topographic barriers and depth [64,65,116]. Dislocation by benthic storms and near-bottom currents has been discussed as potential mechanisms to promote long-distance dispersal in directly developing taxa, but this will probably not apply to this study as abyssal bottom currents are known to be weak in this area [65,116,117]. Furthermore, the disjunct distribution patterns of genotypic clusters, which are represented in our dataset by few individuals only (*E*. aff. *linearis* and *P*. sp.1; S4 Table), may be an artifact resulting from the limited data available (especially between both claims) as well as faunal patchiness [118].

The most common polychaete taxa in our study showing wide distributions were paralacydoniids, goniadids, spionids and opheliids. These taxa cross a range of functional groups, from carnivores to detritivores and exhibit a tremendous range of life-histories, from direct development with no larval dispersal to planktotrophy and teleplanic dispersal. Life-history patterns of deep-sea polychaetes are largely unknown though and most data come from shallow-water species [119–121].

The restricted distributions of some polychaete species may be explained by their development as brooders or having lecithotrophic larvae with limited dispersal capabilities ([122] and references therein, [98]). However, dispersal ability, determined by the life-span of larvae, may not be the sole driver of species ranges. Larvae may not disperse very far but settle in close proximity to parental origins or larvae may have a wider niche than juvenile stages and adults. Furthermore, even adults have the ability to move and hence to disperse. Thus, successful colonization may be governed by ecological constraints on juveniles and adults respectively [95,123].

Our data provide evidence for the presence of cryptic species in isopods and polychaetes (S3 Table, S4 Table). Remarkably, in both isopods and polychaetes, cryptic lineages were often not geographically separated from another, but occurred ‘sympatrically’, that is collected at the same station. The EBS samples over large distances (several kilometers, Table 1), however, and thus may collect animals from different (micro-) habitats characterized by differing environmental conditions. Therefore, it is not clear if lineages actually co-exist spatially or show small-scale patchy distributions.

Lack of phenotypic divergence in species, which are genetically clearly distinct, is known among all metazoan taxa [124]. Two main reasons have been referred to in literature to explain morphological change might not be correlated with genotypic divergence: morphological stasis and nonvisual mating signals [124–126]. Extreme environmental conditions, which apply for deep-sea habitats, might impose stabilizing selection on morphology, preventing [125], or making morphological changes with speciation unnecessary. Furthermore, cryptic species can often be discriminated by differences in mating pheromones. That means organisms need to produce different olfactory signals, but morphological characters do not need to differ appreciably [125]. Such cryptic species might be very common in marine taxa because taxonomists usually lack the knowledge of behavior [127].

Complex interactions across various spatial and temporal scales are probably responsible for the genetic structure observed in both isopods and polychaetes. For example, environmental gradients, such as geographic distance [128], depth- [104,108]), as well as oxygen- [129], temperature- [130] and salinity gradients [131] amongst others, have been discussed as potential factors to promote population differentiation and/or species divergence. In our study, distance and depth are interlinked and may explain genetic variation between distant populations.

### High genetic diversity at local and regional scales

Molecular methods suggest that morphological techniques typically underestimate the number of species and overestimate species ranges in marine habitats [127,132–134].

Depending on the genetic-distance threshold applied, our findings showed a great discrepancy between morphological and genetic diversity of some MOTUs. Genetic diversity (in MOTUs occurring more than once) was overall 2–3 times higher than morphological diversity (Fig. 3). Molecular data provided valuable insights into levels and factors affecting intraspecific divergence, which would not have been picked up by morphological identifications based on the material available for study.

In our study, the degree of faunal turnover, here referred to as the variation in similarity of genotypic clusters between stations, was high and this even at very small (1 km) spatial scales. Within-area differences in faunal similarity were almost as great as between areas. The variation in similarity of MOTUs in the French claim was astonishingly high despite the short distance (1–5 km) between sites. In contrast, the German claim samples were taken much further apart (ranging from 20 to 250 km). At least in the German claim, geographic distance may partly explain levels of faunal variation, while in the French claim the apparent high beta-diversity may be a result of undersampling, i.e. a high number of species with a low number of specimens.

Data on faunal turnover in the deep sea are scarce, but turnover rates seem to be typically very high—ranging between 45–80% over thousands of kilometers—and mainly driven by a large proportion of rare lineages (defined as having low abundance and/or narrow realized distributions) (cf. [13,22,94,102,135]). In high-diversity assemblages—both marine and terrestrial—rare species seem to be common. Rare species may be hard to detect in the deep sea, though, due to its immense size, low sampling effort, and faunal patchiness (cf. [22,118,135]). According to current sampling intensities and the long lists of rare species in our study, it is not possible to determine whether this apparent turnover reflects real endemism or undersampling. Macrofaunal samples were collected with an epibenthic sledge. The sledge recovers non-quantitative samples but provides a much larger sample size than quantitative box-corers. However, samples were still too small to adequately assess connectivity and for making robust assumptions about species-abundance distributions. Consequently, the MOTUs that appear to be rare in our samples may actually exhibit a much wider distribution and/or occur in higher abundances than concluded from the EBS collections. Nevertheless, the high number of singletons obscured the use of traditional statistical tools to identify faunal communities. Furthermore the high frequency of singleton species shows that high replication is required to recover the majority of diversity [6]. In theory, species accumulation curve should reach an asymptote in order to be able to accurately characterize an assemblage [136]. However, due to logistics constrains, we will never be able to collect the great amount of samples necessary to reach an asymptote in the species accumulation curve in the abyss. Although the amount of analyzed polychaete and isopod organisms was quite extensive, the proportion of all collected species probably constitutes less than half of all species represented in the CCZ for real, as indicated from the richness estimators. Collaboration and exchange of taxonomic information as well as molecular barcodes between contractors, could partially overcome this logistic impediment in the CCZ.

Nevertheless, increased sampling effort and stronger replication across different spatial scales may lead to more accurate estimations of geographic ranges and diversity in those genotypic clusters, and will probably reveal many of the rare taxa in our study to be more abundant and/or more widely distributed [22,135,137].

## Conclusion

Reverse taxonomy has proven to be a useful and robust instrument to begin constructing a provisional inventory of the polychaete and isopod fauna and their broad-scale distribution across the CCZ.

However, despite using a relatively large molecular data set investigating long-range distribution of species in polymetallic nodule fields, our results are in some ways still inconclusive. For example, factors and processes leading to a discrepancy between inferred dispersal capabilities and realized distributions in some species (i.e., promoting large-scale distributions in brooders vs. restricted ranges in broadcasters) remain unclear. Moreover, mechanisms mediating population differentiation at local scales need further investigations. Finally, some patterns we observed (e.g. levels of faunal turnover and species ranges) may be the consequence of low sampling effort (as in number of samples and large distance between sites).

Further sampling alongside collection of environmental data will be required for the assessment of natural variability in abundance and diversity across different spatial scales. Information on, for instance, life histories, and ecological constraints of individual species (e.g. based on species distribution models) may help to identify the intrinsic and extrinsic factors limiting dispersal and gene flow and thus geographic range size across the CCZ. Identification of the patterns and processes underlying changes in distribution will lead to progress for conservation management and will provide guidance for conservation planning and design of protected areas.

This study represents only a first step towards understanding diversity of two macrobenthic groups in the CCZ and there are furthermore few comparable data to measure our findings against. However, the high genetic diversity, especially the high proportion of cryptic species even in good dispersers and at very small spatial scales, leads to the assumption that the central Pacific might indeed be listed amongst the World’s most diverse deep-sea ecosystems (cf.[22]).

## Supporting Information

S1 Table Polychaete species IDs and accession numbers for nucleotide sequences retrieved from GenBank. (DOCX)

S2 Table Isopod species IDs and accession numbers for nucleotide sequences retrieved from GenBank. (DOCX)

S3 Table Polychaete MOTUs present in the French and German license areas including morphological determination; la^1^ = license area, g = German license area, f = French license area, seq. ident.^2^ = sequence identity, * = reference sequence. (DOCX)

S4 Table Isopod MOTUs present in the French and German license areas including morphological determination; la^1^ = license area, g = German license area, f = French license area, seq. ident.^2^ = sequence identity,* = reference sequence. (DOCX)
