# Supplementary material for: A Reverse Taxonomic Approach to Assess Macrofaunal Distribution Patterns in Abyssal Pacific Polymetallic Nodule Fields
Source: PLoS One. 2015 Feb 11;10(2):e0117790. doi: 10.1371/journal.pone.0117790 (PMC4324633; doi:10.1371/journal.pone.0117790)
Supplement: S1 Table — (DOCX) [file pone.0117790.s001.docx]

Electronic supplementary material to:

A reverse taxonomic approach to assess macrofaunal distribution patterns in abyssal Pacific polymetallic nodule fields

Annika Janssen^1^*, Stefanie Kaiser^1^, Karin Meißner^2^, Nils Brenke^1^, Lenaick Menot^3^, Pedro Martínez Arbízu^1^

S1 Table: Polychaete species IDs and accession numbers for nucleotide sequences retrieved from GenBank.

Species ID accession number

NB-Po113 KJ736168

EBS47o-Po4 KJ736169

EBS12o-Po23 KJ736170

EBS47o-Po3 KJ736171

EBS47u-Po32 KJ736172

NB-Po152 KJ736173

NB-Po545 KJ736174

EBS61o-Po36 KJ736175

EBS61o-Po116 KJ736176

EBS61o-Po31 KJ736177

EBS61o-Po11 KJ736178

NB-Po169 KJ736179

NB-Po306 KJ736180

NB-Po305 KJ736181

EBS61o-Po77 KJ736182

NB-Po372 KJ736183

NB-Po429 KJ736184

NB-Po373 KJ736185

NB-Po220 KJ736186

MA15 KJ736187

NB-Po578 KJ736188

EBS12o-Po41 KJ736189

NB-Po230 KJ736190

NB-Po571 KJ736191

NB-Po556 KJ736192

EBS47u-Po38 KJ736193

EBS61u-Po4 KJ736194

EBS47u-FPo1 KJ736195

NB-Po591 KJ736196

NB-Po559 KJ736197

NB-Po181 KJ736198

NB-Po180 KJ736199

NB-Po97 KJ736200

MA9 KJ736201

NB-Po421 KJ736202

EBS26o-Po162 KJ736203

NB-Po422 KJ736204

NB-Po375 KJ736205

EBS61o-Po10 KJ736206

EBS61o-Po9 KJ736207

NB-Po292 KJ736208

NB-Po410 KJ736209

NB-Po408 KJ736210

NB-Po138 KJ736211

NB-Po16 KJ736212

NB-Po137 KJ736213

EBS26o-Po9 KJ736214

NB-Po620 KJ736215

NB-Po619 KJ736216

EBS26o-Po07 KJ736217

EBS12o-Po175 KJ736218

NB-Po25 KJ736219

EBS47o-FPo1 KJ736220

MA33 KJ736221

EBS47u-Po9 KJ736222

NB-Po393 KJ736223

NB-Po539 KJ736224

NB-Po392 KJ736225

EBS61o-Po118 KJ736226

NB-Po105 KJ736227

NB-Po341 KJ736228

NB-Po462 KJ736229

NB-Po106 KJ736230

EBS26o-Po54 KJ736231

NB-Po312 KJ736232

MA25 KJ736233

EBS26o-Po196 KJ736234

EBS26o-Po73 KJ736235

EBS26o-Po60 KJ736236

EBS26o-Po53 KJ736237

NB-Po461 KJ736238

NB-Po321 KJ736239

NB-Po622 KJ736240

NB-Po572 KJ736241

NB-Po355 KJ736242

EBS61o-Po119 KJ736243

EBS12o-Po36 KJ736244

EBS47o-Po50 KJ736245

EBS47o-FPo5 KJ736246

EBS26o-Po175 KJ736247

EBS61o-Po44 KJ736248

NB-Po604 KJ736249

EBS26o-Po116 KJ736250

NB-Po549 KJ736251

NB-Po114 KJ736252

EBS26o-Po65 KJ736253

EBS26o-Po63 KJ736254

NB-Po506 KJ736255

EBS61o-Po127 KJ736256

NB-Po521 KJ736257

NB-Po547 KJ736258

NB-Po542 KJ736259

NB-Po203 KJ736260

NB-Po426 KJ736261

EBS47o-Po48 KJ736262

EBS47u-Po18 KJ736263

NB-Po398 KJ736264

EBS26o-Po115 KJ736265

NB-Po223 KJ736266

NB-Po612 KJ736267

NB-Po174 KJ736268

NB-Po475 KJ736269

NB-Po9 KJ736270

EBS26o-Po72 KJ736271

NB-Po115 KJ736272

EBS26o-Po40 KJ736273

EBS26o-Po44 KJ736274

NB-Po88 KJ736275

NB-Po85 KJ736276

NB-Po17 KJ736277

MA29 KJ736278

NB-Po348 KJ736279

NB-Po474 KJ736280

NB-Po603 KJ736281

EBS61o-Po75 KJ736282

EBS26o-Po81 KJ736283

EBS26o-Po68 KJ736284

NB-Po511 KJ736285

MA10 KJ736286

EBS12o-Po67 KJ736287

EBS12o-Po47 KJ736288

NB-Po616 KJ736289

NB-Po510 KJ736290

NB-Po336 KJ736291

NB-Po151 KJ736292

EBS12o-Po28 KJ736293

NB-Po45 KJ736294

NB-Po453 KJ736295

EBS12o-Po46 KJ736296

EBS12o-Po22 KJ736297

NB-Po284 KJ736298

NB-Po153 KJ736299

EBS26o-Po28 KJ736300

EBS12o-Po48 KJ736301

MA16 KJ736302

NB-Po454 KJ736303

EBS26o-Po185 KJ736304

EBS47u-Po21 KJ736305

NB-Po548 KJ736306

NB-Po515 KJ736307

NB-Po331 KJ736308

EBS47o-Po71 KJ736309

NB-Po154 KJ736310

NB-Po50 KJ736311

EBS26o-Po37 KJ736312

EBS47u-Po22 KJ736313

NB-Po134 KJ736314

EBS26o-Po198 KJ736315

EBS47u-Po23 KJ736316

EBS26o-Po112 KJ736317

EBS26o-Po79 KJ736318

NB-Po459 KJ736319

NB-Po13 KJ736320

EBS26o-Po69 KJ736321

EBS26o-Po59 KJ736322

EBS26o-Po18 KJ736323

EBS26o-Po6 KJ736324

EBS47u-Po17 KJ736325

EBS61o-Po122 KJ736326

EBS12o-Po5 KJ736327

NB-Po587 KJ736328

NB-Po557 KJ736329

NB-Po147 KJ736330

EBS26o-Po145 KJ736331

NB-Po308 KJ736332

NB-Po35 KJ736333

EBS47o-Po68 KJ736334

EBS12o-Po70 KJ736335

EBS12o-Po10 KJ736336

NB-Po107 KJ736337

EBS12o-Po3 KJ736338

NB-Po579 KJ736339

MA12 KJ736340

EBS47u-Po39 KJ736341

NB-Po570 KJ736342

EBS47u-Po20 KJ736343

NB-Po582 KJ736344

NB-Po60 KJ736345

NB-Po58 KJ736346

NB-Po508 KJ736347

NB-Po480 KJ736348

NB-Po59 KJ736349

EBS47u-Po2 KJ736350

EBS47u-Po37 KJ736351

NB-Po14 KJ736352

NB-Po358 KJ736353

NB-Po357 KJ736354

EBS12o-Po61 KJ736355

NB-Po232 KJ736356

NB-Po602 KJ736357

NB-Po598 KJ736358

NB-Po484 KJ736359

EBS26o-Po100 KJ736360

EBS12o-Po7 KJ736361

NB-Po108 KJ736362

NB-Po440 KJ736363

EBS26o-Po178 KJ736364

NB-Po109 KJ736365

EBS61o-Po30 KJ736366

EBS26o-Po172 KJ736367

NB-Po201 KJ736368

EBS26o-Po83 KJ736369

EBS26o-Po5 KJ736370

NB-Po278 KJ736371

NB-Po65 KJ736372

NB-Po531 KJ736373

NB-Po91 KJ736374

NB-Po518 KJ736375

NB-Po487 KJ736376

NB-Po92 KJ736377

NB-Po460 KJ736378

NB-Po158 KJ736379

NB-Po217 KJ736380

NB-Po412 KJ736381

NB-Po362 KJ736382

NB-Po363 KJ736383

NB-Po218 KJ736384

NB-Po533 KJ736385

NB-Po529 KJ736386

NB-Po600 KJ736387

NB-Po567 KJ736388

NB-Po437 KJ736389

EBS47o-Po11 KJ736390

NB-Po492 KJ736391

NB-Po213 KJ736392

NB-Po530 KJ736393

NB-Po488 KJ736394

NB-Po593 KJ736395

NB-Po575 KJ736396

NB-Po204 KJ736397

NB-Po369 KJ736398

NB-Po436 KJ736399

NB-Po368 KJ736400

NB-Po205 KJ736401

EBS26o-Po93 KJ736402

NB-Po279 KJ736403

NB-Po159 KJ736404

EBS12o-Po101 KJ736405

NB-Po432 KJ736406

NB-Po418 KJ736407

NB-Po431 KJ736408

EBS26o-Po23 KJ736409

EBS47o-Po64 KJ736410

EBS12o-Po152 KJ736411

NB-Po54 KJ736412

NB-Po339 KJ736413

NB-Po340 KJ736414

EBS61o-Po37 KJ736415

EBS61o-Po32 KJ736416

EBS12o-Po40 KJ736417

EBS61o-Po88 KJ736418

MA11 KJ736419

EBS26o-Po55 KJ736420

EBS26o-Po137 KJ736421

NB-Po11 KJ736422

EBS12u-Po7 KJ736423

NB-Po384 KJ736424

NB-Po12 KJ736425

NB-Po527 KJ736426

EBS47u-Po8 KJ736427

NB-Po385 KJ736428

EBS26o-Po133 KJ736429

EBS26o-Po71 KJ736430

EBS26o-Po96 KJ736431

NB-Po458 KJ736432

NB-Po344 KJ736433

NB-Po102 KJ736434

NB-Po457 KJ736435

NB-Po456 KJ736436

EBS26o-Po195 KJ736437

NB-Po62 KJ736438

EBS40o-Po11 KJ736439

NB-Po345 KJ736440

NB-Po150 KJ736441

NB-Po293 KJ736442

EBS26o-Po188 KJ736443

EBS26o-Po170 KJ736444

NB-Po343 KJ736445

EBS26o-Po183 KJ736446

EBS12o-Po12 KJ736447

EBS47u-Po4 KJ736448

NB-Po288 KJ736449

NB-Po120 KJ736450

NB-Po445 KJ736451

NB-Po156 KJ736452

NB-Po614 KJ736453

NB-Po528 KJ736454

MA3 KJ736455

EBS26o-Po89 KJ736456

EBS26o-Po147 KJ736457

EBS26o-Po134 KJ736458

NB-Po611 KJ736459

EBS47o-Po43 KJ736460

EBS47u-Po28 KJ736461

NB-Po209 KJ736462

NB-Po274 KJ736463

NB-Po391 KJ736464

EBS12o-Po185 KJ736465

NB-Po444 KJ736466

NB-Po390 KJ736467

NB-Po471 KJ736468

EBS61o-Po96 KJ736469

EBS26o-Po122 KJ736470

NB-Po501 KJ736471

NB-Po455 KJ736472

EBS26o-Po109 KJ736473

EBS47o-Po42 KJ736474

NB-Po342 KJ736475

EBS61o-Po1 KJ736476

EBS12o-Po124 KJ736477

EBS61o-Po2 KJ736478

EBS61o-Po3 KJ736479

NB-Po136 KJ736480

NB-Po135 KJ736481

NB-Po524 KJ736482

NB-Po503 KJ736483

NB-Po157 KJ736484

EBS61o-Po21 KJ736485

EBS12o-Po38 KJ736486

NB-Po304 KJ736487

EBS12o-Po9 KJ736488

EBS12o-Po19 KJ736489

NB-Po10 KJ736490

EBS12o-Po35 KJ736491

NB-Po502 KJ736492

EBS26o-Po38 KJ736493

EBS26o-Po22 KJ736494

NB-Po568 KJ736495

NB-Po207 KJ736496

NB-Po424 KJ736497

NB-Po320 KJ736498

NB-Po208 KJ736499

NB-Po618 KJ736500

NB-Po452 KJ736501

NB-Po451 KJ736502

NB-Po489 KJ736503

NB-Po434 KJ736504

EBS26o-Po168 KJ736505

NB-Po450 KJ736506

NB-Po621 KJ736507

NB-Po319 KJ736508

NB-Po111 KJ736509

EBS26o-Po3 KJ736510

NB-Po295 KJ736511

NB-Po48 KJ736512

NB-Po66 KJ736513

NB-Po49 KJ736514

NB-Po224 KJ736515

NB-Po15 KJ736516

EBS26o-Po77 KJ736517

MA38 KJ736518

EBS61o-Po90 KJ736519

NB-Po397 KJ736520

EBS47o-Po60 KJ736521

NB-Po420 KJ736522

EBS47o-Po58 KJ736523

NB-Po282 KJ736524

EBS47o-Po56 KJ736525

EBS47o-Po55 KJ736526

NB-Po419 KJ736527

EBS12o-Po1 KJ736528

EBS61u-Po2 KJ736529

NB-Po381 KJ736530

EBS12u-Po1 KJ736531

NB-Po266 KJ736532

EBS12o-Po54 KJ736533

EBS40o-Po8 KJ736534

NB-Po31 KJ736535

NB-Po477 KJ736536

EBS12o-Po181 KJ736537

NB-Po491 KJ736538

NB-Po513 KJ736539

NB-Po595 KJ736540

NB-Po145 KJ736541

EBS47o-Po66 KJ736542

EBS26o-Po92 KJ736543

EBS26o-Po86 KJ736544

EBS12o-Po32 KJ736545

NB-Po228 KJ736546

EBS12o-Po143 KJ736547

NB-Po179 KJ736548

NB-Po231 KJ736549

EBS12o-Po24 KJ736550

NB-Po550 KJ736551

NB-Po433 KJ736552

NB-Po80 KJ736553

EBS26o-Po35 KJ736554

NB-Po589 KJ736555

NB-Po504 KJ736556

NB-Po411 KJ736557

NB-Po307 KJ736558

NB-Po214 KJ736559

NB-Po494 KJ736560

NB-Po490 KJ736561

NB-Po509 KJ736562

NB-Po387 KJ736563

EBS12o-Po8 KJ736564

NB-Po427 KJ736565

EBS26o-Po80 KJ736566

NB-Po607 KJ736567

NB-Po523 KJ736568

NB-Po19 KJ736569

NB-Po386 KJ736570

NB-Po24 KJ736571

EBS26o-Po33 KJ736572

NB-Po67 KJ736573

EBS12o-Po65 KJ736574

NB-Po20 KJ736575

EBS12o-Po52 KJ736576

NB-Po61 KJ736577

NB-Po535 KJ736578

NB-Po171 KJ736579

NB-Po141 KJ736580

EBS47o-Po63 KJ736581

NB-Po555 KJ736582

NB-Po438 KJ736583

EBS12o-Po64 KJ736584

NB-Po534 KJ736585

NB-Po483 KJ736586

NB-Po210 KJ736587

NB-Po271 KJ736588

EBS47o-Po61 KJ736589

NB-Po569 KJ736590

EBS47u-Po33 KJ736591

NB-Po289 KJ736592

NB-Po447 KJ736593

NB-Po290 KJ736594

EBS12o-Po17 KJ736595

NB-Po190 KJ736596

EBS12o-Po59 KJ736597

EBS12o-Po184 KJ736598

EBS12o-Po25 KJ736599

NB-Po540 KJ736600

EBS12o-Po151 KJ736601

NB-Po189 KJ736602

NB-Po225 KJ736603

MA8 KJ736604

EBS12o-Po154 KJ736605

NB-Po70 KJ736606

MA37 KJ736607

MA26 KJ736608

EBS47u-Po10 KJ736609

EBS61o-Po5 KJ736610

NB-Po117 KJ736611

NB-Po69 KJ736612

NB-Po116 KJ736613

MA21 KJ736614

EBS47o-Po67 KJ736615

NB-Po296 KJ736616

NB-Po297 KJ736617

EBS47u-Po40 KJ736618

EBS26o-Po64 KJ736619

EBS12o-Po34 KJ736620

EBS26o-Po193 KJ736621

NB-Po68 KJ736622

EBS26o-Po200 KJ736623

MA28 KJ736624

EBS47u-Po30 KJ736625

EBS12o-Po13 KJ736626

NB-Po350 KJ736627

EBS61o-Po17 KJ736628

EBS12o-Po107 KJ736629

NB-Po482 KJ736630

NB-Po407 KJ736631

EBS40o-Po10 KJ736632

NB-Po351 KJ736633

NB-Po500 KJ736634

NB-Po89 KJ736635

EBS61o-Po16 KJ736636

EBS61o-Po14 KJ736637

EBS12o-Po6 KJ736638

NB-Po497 KJ736639

NB-Po346 KJ736640

MA36 KJ736641

NB-Po347 KJ736642

NB-Po127 KJ736643

NB-Po99 KJ736644

NB-Po388 KJ736645

EBS26o-Po142 KJ736646

EBS12o-Po161 KJ736647

NB-Po389 KJ736648

NB-Po333 KJ736649

NB-Po98 KJ736650

NB-Po64 KJ736651

EBS12o-Po37 KJ736652

NB-Po417 KJ736653

NB-Po416 KJ736654

EBS12o-Po57 KJ736655

NB-Po291 KJ736656

NB-Po601 KJ736657

NB-Po301 KJ736658

NB-Po512 KJ736659

NB-Po498 KJ736660

EBS26o-Po98 KJ736661

NB-Po44 KJ736662

NB-Po558 KJ736663

NB-Po519 KJ736664

NB-Po493 KJ736665

EBS26o-Po103 KJ736666

NB-Po566 KJ736667

NB-Po565 KJ736668

NB-Po63 KJ736669

NB-Po300 KJ736670

NB-Po379 KJ736671

EBS26o-Po62 KJ736672

EBS47o-FPo6 KJ736673

EBS47o-FPo4 KJ736674

EBS26o-Po140 KJ736675

NB-Po428 KJ736676

NB-Po541 KJ736677

NB-Po517 KJ736678

EBS47o-FPo7 KJ736679

EBS26o-Po173 KJ736680

EBS61o-Po98 KJ736681

EBS61o-Po97 KJ736682

NB-Po581 KJ736683

NB-Po55 KJ736684

EBS12o-Po66 KJ736685

EBS47o-Po22 KJ736686

NB-Po255 KJ736687

NB-Po366 KJ736688

EBS61u-UPo5 KJ736689

EBS47u-Po29 KJ736690

EBS47o-Po21 KJ736691

EBS47o-Po19 KJ736692

EBS47o-Po18 KJ736693

EBS12o-Po178 KJ736694

NB-Po443 KJ736695

NB-Po367 KJ736696

MA7 KJ736697

NB-Po254 KJ736698

EBS47u-Po36 KJ736699

MA20 KJ736700

NB-Po590 KJ736701

EBS61u-UPo4 KJ736702

NB-Po72 KJ736703

MA19 KJ736704

EBS12o-Po190 KJ736705

NB-Po441 KJ736706

NB-Po177 KJ736707

EBS26o-Po19 KJ736708

NB-Po140 KJ736709

EBS47u-Po13 KJ736710

NB-Po406 KJ736711

EBS12o-Po43 KJ736712

NB-Po287 KJ736713

NB-Po356 KJ736714

EBS61u-Po9 KJ736715

EBS61o-Po115 KJ736716

EBS61o-Po103 KJ736717

EBS61o-Po123 KJ736718

EBS47o-FPo9 KJ736719

EBS61o-Po130 KJ736720

NB-Po544 KJ736721

EBS61o-Po117 KJ736722

EBS61o-Po128 KJ736723
