# Supplementary material for: A Reverse Taxonomic Approach to Assess Macrofaunal Distribution Patterns in Abyssal Pacific Polymetallic Nodule Fields
Source: PLoS One. 2015 Feb 11;10(2):e0117790. doi: 10.1371/journal.pone.0117790 (PMC4324633; doi:10.1371/journal.pone.0117790)
Supplement: S2 Table — (DOCX) [file pone.0117790.s002.docx]

Electronic supplementary material to:

A reverse taxonomic approach to assess macrofaunal distribution patterns in abyssal Pacific polymetallic nodule fields

Annika Janssen^1^*, Stefanie Kaiser^1^, Karin Meißner^2^, Nils Brenke^1^, Lenaick Menot^3^, Pedro Martínez Arbízu^1^

S2 Table: Isopod species IDs and accession numbers for nucleotide sequences retrieved from GenBank.

Species ID accession number

NB-Iso397 KJ736018

NB-Iso87 KJ736019

NB-Iso38 KJ736020

NB-Iso56 KJ736021

NB-Iso226 KJ736022

NB-Iso236A KJ736023

NB-Iso221 KJ736024

NB-Iso35 KJ736025

NB-Iso380 KJ736026

NB-Iso31 KJ736027

NB-Iso122 KJ736028

NB-Iso136 KJ736029

NB-Iso216 KJ736030

NB-Iso228 KJ736031

NB-Iso90 KJ736032

NB-Iso36#06 KJ736033

NB-Iso386 KJ736034

NB-Iso86 KJ736035

NB-Iso82 KJ736036

NB-Iso83 KJ736037

NB-Iso451 KJ736038

NB-Iso32 KJ736039

NB-Iso89 KJ736040

NB-Iso114 KJ736041

NB-Iso230 KJ736042

NB-Iso236b KJ736043

NB-Iso467 KJ736044

NB-Iso466 KJ736045

NB-Iso468 KJ736046

NB-Iso396 KJ736047

NB-Iso406 KJ736048

NB-Iso455 KJ736049

NB-Iso33 KJ736050

NB-Iso34 KJ736051

NB-Iso281 KJ736052

NB-Iso307 KJ736053

NB-Iso303 KJ736054

NB-Iso310 KJ736055

NB-Iso405 KJ736056

NB-Iso456 KJ736057

NB-Iso40 KJ736058

NB-Iso239 KJ736059

NB-Iso37 KJ736060

NB-Iso117 KJ736061

NB-Iso46 KJ736062

NB-Iso108 KJ736063

NB-Iso439 KJ736064

NB-Iso74 KJ736065

NB-Iso75 KJ736066

NB-Iso8 KJ736067

NB-Iso4 KJ736068

NB-Iso76 KJ736069

NB-Iso95 KJ736070

NB-Iso193 KJ736071

NB-Iso515 KJ736072

NB-Iso204 KJ736073

NB-Iso197 KJ736074

NB-Iso264 KJ736075

NB-Iso192 KJ736076

NB-Iso210 KJ736077

NB-Iso194 KJ736078

NB-Iso441 KJ736079

NB-Iso195 KJ736080

NB-Iso233 KJ736081

NB-Iso343 KJ736082

NB-Iso345 KJ736083

NB-Iso347 KJ736084

NB-Iso341 KJ736085

NB-Iso1 KJ736086

NB-Iso3 KJ736087

NB-Iso2 KJ736088

NB-Iso5 KJ736089

NB-Iso344 KJ736090

NB-Iso250 KJ736091

NB-Iso355 KJ736092

NB-Iso256 KJ736093

NB-Iso253 KJ736094

NB-Iso458 KJ736095

NB-Iso348 KJ736096

NB-Iso284 KJ736097

NB-Iso285 KJ736098

NB-Iso99 KJ736099

NB-Iso463 KJ736100

NB-Iso70 KJ736101

NB-Iso290 KJ736102

NB-Iso330 KJ736103

NB-Iso68 KJ736104

NB-Iso20 KJ736105

NB-Iso98 KJ736106

NB-Iso445 KJ736107

NB-Iso263 KJ736108

NB-Iso245 KJ736109

NB-Iso243 KJ736110

NB-Iso454 KJ736111

NB-Iso30 KJ736112

NB-Iso52 KJ736113

NB-Iso71 KJ736114

NB-Iso486 KJ736115

NB-Iso425 KJ736116

NB-Iso444 KJ736117

NB-Iso460 KJ736118

NB-Iso462 KJ736119

NB-Iso353 KJ736120

NB-Iso354 KJ736121

NB-Iso29 KJ736122

NB-Iso514 KJ736123

NB-Iso512 KJ736124

NB-Iso43 KJ736125

NB-Iso81 KJ736126

NB-Iso78 KJ736127

NB-Iso64 KJ736128

NB-Iso41 KJ736129

NB-Iso79 KJ736130

NB-Iso112 KJ736131

NB-Iso168 KJ736132

NB-Iso170 KJ736133

NB-Iso508 KJ736134

NB-Iso443 KJ736135

NB-Iso100 KJ736136

NB-Iso105 KJ736137

NB-Iso102 KJ736138

NB-Iso104 KJ736139

NB-Iso268 KJ736140

NB-Iso356 KJ736141

NB-Iso101 KJ736142

NB-Iso103 KJ736143

NB-Iso267 KJ736144

NB-Iso448 KJ736145

NB-Iso44 KJ736146

NB-Iso42 KJ736147

NB-Iso45 KJ736148

NB-Iso244 KJ736149

NB-Iso49 KJ736150

NB-Iso499 KJ736151

NB-Iso111 KJ736152

NB-Iso276 KJ736153

NB-Iso47 KJ736154

NB-Iso241 KJ736155

NB-Iso497 KJ736156

NB-Iso339 KJ736157

NB-Iso337 KJ736158

NB-Iso282 KJ736159

NB-Iso279 KJ736160

NB-Iso461 KJ736161

NB-Iso242 KJ736162

NB-Iso273 KJ736163

NB-Iso280 KJ736164

NB-Iso283 KJ736165

NB-Iso234 KJ736166

NB-Iso446 KJ736167
