# Supplementary material for: A Reverse Taxonomic Approach to Assess Macrofaunal Distribution Patterns in Abyssal Pacific Polymetallic Nodule Fields
Source: PLoS One. 2015 Feb 11;10(2):e0117790. doi: 10.1371/journal.pone.0117790 (PMC4324633; doi:10.1371/journal.pone.0117790)
Supplement: S3 Table — (DOCX) [file pone.0117790.s003.docx]

Electronic supplementary material to:

A reverse taxonomic approach to assess macrofaunal distribution patterns in abyssal Pacific polymetallic nodule fields

Annika Janssen^1^*, Stefanie Kaiser^1^, Karin Meißner^2^, Nils Brenke^1^, Lenaick Menot^3^, Pedro Martínez Arbízu^1^

S3 Table: Polychaete MOTUs present in the French and German license area including morphological determination; la^1^= license area, g=German license area, f = French license area, seq. ident.^2^ = sequence identity, * = reference sequence.

| **cls-no** | **species ID** | **EBS#** | **la^1^** | **family** | ***genus*** | ***species*** | **seq. ident.^2^**  **(in %)** |
| --- | --- | --- | --- | --- | --- | --- | --- |
| 0 | \| NB-Po366 \| 33 \| g \| Paralacydoniidae \| *Paralacydonia* \| cf. weberi \| * \| \| --- \| --- \| --- \| --- \| --- \| --- \| --- \| \| EBS61u-ÜPo5 \| 61 \| g \| Paralacydoniidae \| *Paralacydonia* \| cf. weberi \| 99 \| \| EBS47u-Po29 \| 47 \| g \| Paralacydoniidae \| *Paralacydonia* \| cf. weberi \| 99 \| \| EBS47o-Po21 \| 47 \| g \| Paralacydoniidae \| *Paralacydonia* \| cf. weberi \| 99 \| \| EBS47o-Po19 \| 47 \| g \| Paralacydoniidae \| *Paralacydonia* \| cf. weberi \| 99 \| \| EBS47o-Po18 \| 47 \| g \| Paralacydoniidae \| *Paralacydonia* \| cf. weberi \| 99 \| \| EBS12o-Po178 \| 12 \| g \| Paralacydoniidae \| *Paralacydonia* \| cf. weberi \| 99 \| \| NB-Po443 \| 51 \| g \| Paralacydoniidae \| *Paralacydonia* \| cf. weberi \| 99 \| \| NB-Po367 \| 33 \| g \| Paralacydoniidae \| *Paralacydonia* \| cf. weberi \| 99 \| \| MA7 \| 12 \| g \| Paralacydoniidae \| *Paralacydonia* \| cf. weberi \| 97 \| \| NB-Po254 \| 67 \| f \| Paralacydoniidae \| *Paralacydonia* \| cf. weberi \| 98 \| \| EBS47u-Po36 \| 47 \| g \| Paralacydoniidae \| *Paralacydonia* \| cf. weberi \| 98 \| \| MA20 \| 26 \| g \| Paralacydoniidae \| *Paralacydonia* \| cf. weberi \| 97 \| \| NB-Po590 \| 101 \| f \| Paralacydoniidae \| *Paralacydonia* \| cf. weberi \| 98 \| \| EBS61u-ÜPo4 \| 61 \| g \| Paralacydoniidae \| *Paralacydonia* \| cf. weberi \| 98 \| \| MA19 \| 12 \| g \| Paralacydoniidae \| *Paralacydonia* \| cf. weberi \| 98 \| \| EBS12o-Po190 \| 12 \| g \| Paralacydoniidae \| *Paralacydonia* \| cf. weberi \| 98 \| \| NB-Po441 \| 51 \| g \| Paralacydoniidae \| *Paralacydonia* \| cf. weberi \| 98 \| \| MA18 \| 26 \| g \| Paralacydoniidae \| *Paralacydonia* \| cf. weberi \| 99 \| \| EBS12o-Po66 \| 12 \| g \| Paralacydoniidae \| *Paralacydonia* \| cf. weberi \| 99 \| \| EBS47o-Po22 \| 47 \| g \| Paralacydoniidae \| *Paralacydonia* \| cf. weberi \| 99 \| \| NB-Po255 \| 67 \| g \| Paralacydoniidae \| *Paralacydonia* \| cf. weberi \| 99 \| \| NB-Po72 \| 06 \| g \| Paralacydoniidae \| *Paralacydonia* \| cf. weberi \| 98 \| | | | | | | |
| 2 | \| EBS12o-Po40 \| 12 \| g \| Goniadidae \| *Bathyglycinde* \| cf.*profunda* \| 98 \| \| --- \| --- \| --- \| --- \| --- \| --- \| --- \| \| EBS61o-Po88 \| 61 \| g \| Goniadidae \| *Bathyglycinde* \| cf.*profunda* \| * \| \| MA11 \| 12 \| g \| Goniadidae \| *Bathyglycinde* \| cf.*profunda* \| 99 \| \| EBS26o-Po55 \| 26 \| g \| Goniadidae \| *Bathyglycinde* \| cf.*profunda* \| 98 \| \| EBS26o-Po137 \| 26 \| g \| Goniadidae \| *Bathyglycinde* \| cf.*profunda* \| 98 \| \| NB-Po11 \| 06 \| g \| Goniadidae \| *Bathyglycinde* \| cf.*profunda* \| 98 \| \| EBS12u-Po7 \| 12 \| g \| Goniadidae \| *Bathyglycinde* \| cf.*profunda* \| 98 \| \| NB-Po384 \| 33 \| g \| Goniadidae \| *Bathyglycinde* \| cf.*profunda* \| 98 \| \| NB-Po12 \| 12 \| g \| Goniadidae \| *Bathyglycinde* \| cf.*profunda* \| 98 \| \| NB-Po527 \| 107 \| f \| Goniadidae \| *Bathyglycinde* \| cf.*profunda* \| 99 \| \| EBS47u-Po8 \| 47 \| g \| Goniadidae \| *Bathyglycinde* \| cf.*profunda* \| 99 \| \| NB-Po385 \| 33 \| g \| Goniadidae \| *Bathyglycinde* \| cf.*profunda* \| 99 \| \| EBS26o-Po133 \| 26 \| g \| Goniadidae \| *Bathyglycinde* \| cf.*profunda* \| 99 \| \| EBS26o-Po71 \| 26 \| g \| Goniadidae \| *Bathyglycinde* \| cf.*profunda* \| 99 \| \| EBS26o-Po96 \| 26 \| g \| Goniadidae \| *Bathyglycinde* \| cf.*profunda* \| 99 \| | | | | | | |
| 4 | \| NB-Po190 \| 73 \| f \| Sigalionidae \| ?*Leanira* \| sp. 1 \| * \| \| --- \| --- \| --- \| --- \| --- \| --- \| --- \| \| EBS12o-Po59 \| 12 \| g \| Sigalionidae \| ?*Leanira* \| sp. 1 \| 99 \| \| EBS12o-Po184 \| 12 \| g \| Sigalionidae \| ?*Leanira* \| sp. 1 \| 99 \| \| EBS12o-Po25 \| 12 \| g \| Sigalionidae \| ?*Leanira* \| sp. 1 \| 99 \| \| NB-Po540 \| 107 \| f \| Sigalionidae \| ?*Leanira* \| sp. 1 \| 99 \| \| EBS12o-Po151 \| 12 \| g \| Sigalionidae \| ?*Leanira* \| sp. 1 \| 99 \| \| NB-Po189 \| 73 \| f \| Sigalionidae \| ?*Leanira* \| sp. 1 \| 99 \| \| NB-Po225 \| 80 \| f \| Sigalionidae \| ?*Leanira* \| sp. 1 \| 99 \| \| MA8 \| 12 \| g \| Sigalionidae \| ?*Leanira* \| sp. 1 \| 99 \| \| EBS12o-Po154 \| 12 \| g \| Sigalionidae \| ?*Leanira* \| sp. 1 \| 99 \| \| EBS12o-Po17 \| 12 \| g \| Sigalionidae \| ?*Leanira* \| sp. 1 \| 99 \| | | | | | | |
|  | NB-Po207 | 80 | f | Spionidae | *Prionospio* | sp. 1 | * |
|  | NB-Po424 | 51 | g | Spionidae | *Prionospio* | sp. 1 | 98 |
|  | NB-Po320 | 43 | g | Spionidae | *Prionospio* | sp. 1 | 98 |
|  | NB-Po208 | 80 | f | Spionidae | *Prionospio* | sp. 1 | 98 |
| 7 | NB-Po618 | 107 | f | Spionidae | *Prionospio* | sp. 1 | 99 |
|  | NB-Po452 | 06 | g | Spionidae | *Prionospio* | sp. 1 | 99 |
|  | NB-Po451 | 06 | g | Spionidae | *Prionospio* | sp. 1 | 98 |
|  | NB-Po489 | 101 | f | Spionidae | *Prionospio* | sp. 1 | 98 |
|  | NB-Po434 | 51 | g | Spionidae | *Prionospio* | sp. 1 | 99 |
|  | EBS26o-Po168 | 26 | g | Spionidae | *Prionospio* | sp. 1 | 99 |
| 8 | \| NB-Po17 \| 06 \| g \| Cirratulidae \| *Chaetozone* \| cf. *corona* \| * \| \| --- \| --- \| --- \| --- \| --- \| --- \| --- \| \| MA29 \| 26 \| g \| Cirratulidae \| *Chaetozone* \| cf. *corona* \| 98 \| \| NB-Po348 \| 43 \| g \| Cirratulidae \| *Chaetozone* \| cf. *corona* \| 99 \| \| NB-Po474 \| 06 \| g \| Cirratulidae \| *Chaetozone* \| cf. *corona* \| 99 \| \| NB-Po603 \| 107 \| f \| Cirratulidae \| *Chaetozone* \| cf. *corona* \| 99 \| \| EBS61o-Po75 \| 61 \| g \| Cirratulidae \| *Chaetozone* \| cf. *corona* \| 99 \| \| EBS26o-Po81 \| 26 \| g \| Cirratulidae \| *Chaetozone* \| cf. *corona* \| 99 \| \| EBS26o-Po68 \| 26 \| g \| Cirratulidae \| *Chaetozone* \| cf. *corona* \| 99 \| | | | | | | |
| 9 | \| NB-Po65 \| 06 \| g \| Opheliidae \| *Ophelina* \| sp. 2 \| * \| \| --- \| --- \| --- \| --- \| --- \| --- \| --- \| \| NB-Po531 \| 107 \| f \| Opheliidae \| *Ophelina* \| sp. 2 \| 99 \| \| NB-Po91 \| 16 \| g \| Opheliidae \| *Ophelina* \| sp. 2 \| 99 \| \| NB-Po518 \| 107 \| f \| Opheliidae \| *Ophelina* \| sp. 2 \| 98 \| \| NB-Po487 \| 101 \| f \| Opheliidae \| *Ophelina* \| sp. 2 \| 98 \| \| NB-Po92 \| 16 \| g \| Opheliidae \| *Ophelina* \| sp. 2 \| 98 \| \| NB-Po278 \| 43 \| g \| Opheliidae \| *Ophelina* \| sp. 2 \| 97 \| \| EBS26o-Po83 \| 26 \| g \| Opheliidae \| *Ophelina* \| sp. 2 \| 97 \| \| EBS26o-Po5 \| 26 \| g \| Opheliidae \| *Ophelina* \| sp. 2 \| 97 \| | | | | | | |
| 10 | \| EBS12o-Po8 \| 12 \| g \| Spionidae \| *Laonice* \| sp. 1 \| * \| \| --- \| --- \| --- \| --- \| --- \| --- \| --- \| \| NB-Po427 \| 51 \| g \| Spionidae \| *Laonice* \| sp. 1 \| 99 \| \| EBS26o-Po80 \| 26 \| g \| Spionidae \| *Laonice* \| sp. 1 \| 99 \| \| NB-Po607 \| 107 \| f \| Spionidae \| *Laonice* \| sp. 1 \| 99 \| \| NB-Po523 \| 107 \| f \| Spionidae \| *Laonice* \| sp. 1 \| 99 \| \| NB-Po19 \| 06 \| g \| Spionidae \| *Laonice* \| sp. 1 \| 99 \| \| NB-Po386 \| 33 \| g \| Spionidae \| *Laonice* \| sp. 1 \| 99 \| \| NB-Po387 \| 33 \| g \| Spionidae \| *Laonice* \| sp. 1 \| 98 \| | | | | | | |
| 12 | \| NB-Po582 \| 101 \| f \| Magelonidae \| *Octomagelona* \| sp. 1 \| * \| \| --- \| --- \| --- \| --- \| --- \| --- \| --- \| \| NB-Po60 \| 06 \| g \| Magelonidae \| *Octomagelona* \| sp. 1 \| 98 \| \| NB-Po58 \| 06 \| g \| Magelonidae \| *Octomagelona* \| sp. 1 \| 98 \| \| NB-Po508 \| 101 \| f \| Magelonidae \| *Octomagelona* \| sp. 1 \| 98 \| \| NB-Po480 \| 06 \| g \| Magelonidae \| *Octomagelona* \| sp. 1 \| 98 \| \| NB-Po59 \| 06 \| g \| Magelonidae \| *Octomagelona* \| sp. 1 \| 98 \| \| EBS47u-Po2 \| 47 \| g \| Magelonidae \| *Octomagelona* \| sp. 1 \| 98 \| | | | | | | |
| 14 | \| NB-Po555 \| 107 \| f \| Opheliidae \| *Ophelina* \| sp. 1 \| * \| \| --- \| --- \| --- \| --- \| --- \| --- \| --- \| \| NB-Po438 \| 51 \| g \| Opheliidae \| *Ophelina* \| sp. 1 \| 99 \| \| EBS12o-Po64 \| 12 \| g \| Opheliidae \| *Ophelina* \| sp. 1 \| 98 \| \| NB-Po534 \| 107 \| f \| Opheliidae \| *Ophelina* \| sp. 1 \| 99 \| \| NB-Po483 \| 101 \| f \| Opheliidae \| *Ophelina* \| sp. 1 \| 99 \| \| NB-Po210 \| 80 \| f \| Opheliidae \| *Ophelina* \| sp. 1 \| 99 \| \| NB-Po271 \| 80 \| f \| Opheliidae \| *Ophelina* \| sp. 1 \| 99 \| | | | | | | |
| 15 | \| NB-Po223 \| 80 \| f \| Cirratulidae \|  \| sp.2 \| * \| \| --- \| --- \| --- \| --- \| --- \| --- \| --- \| \| NB-Po612 \| 107 \| f \| Cirratulidae \|  \| sp.2 \| 99 \| \| NB-Po174 \| 73 \| f \| Cirratulidae \|  \| sp.2 \| 99 \| \| NB-Po475 \| 06 \| g \| Cirratulidae \|  \| sp.2 \| 98 \| \| NB-Po9 \| 06 \| g \| Cirratulidae \|  \| sp.2 \| 99 \| \| EBS26o-Po115 \| 26 \| g \| Cirratulidae \|  \| sp.2 \| 99 \| | | | | | | |
| 16 | \| NB-Po600 \| 107 \| f \| Opheliidae \|  \| sp.2 \| * \| \| --- \| --- \| --- \| --- \| --- \| --- \| --- \| \| NB-Po567 \| 107 \| f \| Opheliidae \|  \| sp.2 \| 98 \| \| NB-Po437 \| 51 \| g \| Opheliidae \|  \| sp.2 \| 98 \| \| EBS47o-Po11 \| 47 \| g \| Opheliidae \|  \| sp.2 \| 98 \| \| NB-Po492 \| 101 \| f \| indet \|  \|  \| 98 \| \| NB-Po213 \| 80 \| f \| Opheliidae \|  \| sp.2 \| 98 \| | | | | | | |
| 17 | \| NB-Po436 \| 51 \| g \| Opheliidae \|  \| sp.2 \| * \| \| --- \| --- \| --- \| --- \| --- \| --- \| --- \| \| NB-Po368 \| 33 \| g \| Opheliidae \|  \| sp.2 \| 99 \| \| NB-Po205 \| 80 \| f \| Opheliidae \|  \| sp.2 \| 99 \| \| EBS26o-Po93 \| 26 \| g \| Opheliidae \|  \| sp.2 \| 99 \| \| NB-Po279 \| 43 \| g \| Opheliidae \|  \| sp.2 \| 99 \| \| NB-Po159 \| 33 \| g \| Spionidae \|  \| sp.1 \| 99 \| | | | | | | |
| 22 | \| NB-Po569 \| 107 \| f \| Pholoidae \|  \| sp.1 \| * \| \| --- \| --- \| --- \| --- \| --- \| --- \| --- \| \| EBS47u-Po33 \| 47 \| g \| Pholoidae \|  \| sp.1 \| 98 \| \| NB-Po289 \| 43 \| g \| Pholoidae \|  \| sp.1 \| 99 \| \| NB-Po447 \| 51 \| g \| Pholoidae \|  \| sp.1 \| 99 \| \| NB-Po290 \| 43 \| g \| Pholoidae \|  \| sp.1 \| 99 \| \| EBS47o-Po61 \| 47 \| g \| Pholoidae \|  \| sp.1 \| 99 \| | | | | | | |
| 24 | \| NB-Po541 \| 107 \| f \| Hesionidae \|  \| sp.1 \| * \| \| --- \| --- \| --- \| --- \| --- \| --- \| --- \| \| NB-Po517 \| 101 \| f \| Hesionidae \|  \| sp.1 \| 99 \| \| EBS47o-FPo7 \| 47 \| g \| Hesionidae \|  \| sp.1 \| 99 \| \| EBS26o-Po173 \| 26 \| g \| Hesionidae \|  \| sp.1 \| 99 \| \| NB-Po428 \| 51 \| g \| Hesionidae \|  \| sp.1 \| 99 \| | | | | | | |
| 26 | \| NB-Po506 \| 101 \| f \| Polychaeta indet. \|  \| sp.1 \| * \| \| --- \| --- \| --- \| --- \| --- \| --- \| --- \| \| EBS61o-Po127 \| 61 \| g \| Polychaeta indet. \|  \| sp.1 \| 99 \| \| NB-Po521 \| 107 \| f \| Polychaeta indet. \|  \| sp.1 \| 99 \| \| NB-Po547 \| 107 \| f \| Polychaeta indet. \|  \| sp.1 \| 98 \| \| NB-Po542 \| 107 \| f \| Polychaeta indet. \|  \| sp.1 \| 99 \| | | | | | | |
| 29 | \| NB-Po450 \| 06 \| g \| Spionidae \| *Prionospio* \| sp.1 \| * \| \| --- \| --- \| --- \| --- \| --- \| --- \| --- \| \| NB-Po621 \| 107 \| f \| Spionidae \| *Prionospio* \| sp.1 \| 99 \| \| NB-Po111 \| 16 \| g \| Spionidae \| *Prionospio* \| sp.1 \| 99 \| \| EBS26o-Po3 \| 26 \| g \| Spionidae \| *Prionospio* \| sp.1 \| 99 \| \| NB-Po319 \| 43 \| g \| Spionidae \| *Prionospio* \| sp.1 \| 99 \| | | | | | | |
| 31 | \| NB-Po504 \| 101 \| f \| Poecilochaetidae \| *Poecilochaetus* \| sp.1 \| * \| \| --- \| --- \| --- \| --- \| --- \| --- \| --- \| \| NB-Po411 \| 51 \| g \| Poecilochaetidae \| *Poecilochaetus* \| sp.1 \| 100 \| \| NB-Po307 \| 43 \| g \| Poecilochaetidae \| *Poecilochaetus* \| sp.1 \| 100 \| \| NB-Po214 \| 80 \| f \| Poecilochaetidae \| *Poecilochaetus* \| sp.1 \| 100 \| \| NB-Po589 \| 101 \| f \| Poecilochaetidae \| *Poecilochaetus* \| sp.1 \| 99 \| | | | | | | |
| 32 | \| NB-Po497 \| 101 \| f \| Ampharetidae \|  \| sp.1 \| * \| \| --- \| --- \| --- \| --- \| --- \| --- \| --- \| \| NB-Po346 \| 06 \| g \| Ampharetidae \|  \| sp.1 \| 98 \| \| NB-Po347 \| 06 \| g \| Ampharetidae \|  \| sp.1 \| 98 \| \| EBS12o-Po6 \| 12 \| g \| Ampharetidae \|  \| sp.1 \| 98 \| \| MA36 \| 26 \| g \| Ampharetidae \|  \| sp.1 \| 99 \| \| NB-Po127 \| 33 \| g \| Ampharetidae \|  \| sp.1 \| 99 \| \| EBS61o-Po14 \| 61 \| g \| Ampharetidae \|  \| sp.1 \| 98 \| | | | | | | |
| 33 | \| NB-Po601 \| 107 \| f \| Spionidae \| *Spiophanes* \| sp.1 \| * \| \| --- \| --- \| --- \| --- \| --- \| --- \| --- \| \| NB-Po301 \| 43 \| g \| Spionidae \| *Spiophanes* \| sp.1 \| 98 \| \| NB-Po512 \| 101 \| f \| Spionidae \| *Spiophanes* \| sp.1 \| 98 \| \| NB-Po498 \| 101 \| f \| Spionidae \| *Spiophanes* \| sp.1 \| 97 \| \| EBS26o-Po98 \| 26 \| g \| Spionidae \| *Spiophanes* \| sp.1 \| 97 \| | | | | | | |
|  | NB-Po412 | 51 | g | Opheliidae |  | sp.3 | * |
| 37 | NB-Po362 | 33 | g | Opheliidae |  | sp.3 | 99 |
|  | NB-Po363 | 33 | g | Opheliidae |  | sp.3 | 99 |
|  | NB-Po218 | 80 | f | Opheliidae |  | sp.3 | 99 |
| 39 | \| NB-Po524 \| 107 \| f \| Amphinomidea \| *Chloeia* \| sp.1 \| * \| \| --- \| --- \| --- \| --- \| --- \| --- \| --- \| \| NB-Po503 \| 101 \| f \| Amphinomidea \| *Chloeia* \| sp.1 \| 99 \| \| NB-Po157 \| 33 \| g \| Amphinomidea \| *Chloeia* \| sp.1 \| 98 \| \| EBS61o-Po21 \| 61 \| g \| Amphinomidea \| *Chloeia* \| sp.1 \| 98 \| | | | | | | |
| 42 | \| EBS61o-Po130 \| 61 \| g \| Polychaeta indet \|  \| sp.1 \| 97 \| \| --- \| --- \| --- \| --- \| --- \| --- \| --- \| \| NB-Po544 \| 107 \| f \| Polychaeta indet \|  \| sp.1 \| * \| \| EBS61o-Po117 \| 61 \| g \| Polychaeta indet \|  \| sp.1 \| 98 \| \| EBS61o-Po128 \| 61 \| g \| Polychaeta indet \|  \| sp.1 \| 98 \| | | | | | | |
| 49 | \| EBS12o-Po47 \| 12 \| g \| Acrocirridae \|  \| sp.1 \| 99 \| \| --- \| --- \| --- \| --- \| --- \| --- \| --- \| \| NB-Po616 \| 107 \| f \| Acrocirridae \|  \| sp.1 \| * \| \| NB-Po510 \| 101 \| f \| Acrocirridae \|  \| sp.1 \| 97 \| | | | | | | |
| 57 | \| NB-Po220 \| 80 \| f \| Paraonidae \|  \| sp.1 \| * \| \| --- \| --- \| --- \| --- \| --- \| --- \| --- \| \| MA15 \| 12 \| g \| Paraonidae \|  \| sp.1 \| 99 \| | | | | | | |
| 83 | \| NB-Po550 \| 107 \| f \| Spionidae \| *Laonice* \| sp 2 \| * \| \| --- \| --- \| --- \| --- \| --- \| --- \| --- \| \| NB-Po433 \| 51 \| g \| Spionidae \| *Prionospio* \| sp.2 \| 99 \| | | | | | | |
| 88 | \| NB-Po500 \| 101 \| f \| Ampharetidae \|  \| sp.2 \| * \| \| --- \| --- \| --- \| --- \| --- \| --- \| --- \| \| NB-Po89 \| 16 \| g \| Ampharetidae \|  \| sp.2 \| 99 \| | | | | | | |
| 94 | \| NB-Po231 \| 80 \| f \| Polychaeta indet \|  \| sp.2 \| * \| \| --- \| --- \| --- \| --- \| --- \| --- \| --- \| \| EBS12o-Po24 \| 12 \| g \| Polychaeta indet \|  \| sp.2 \| 100 \| | | | | | | |
