# Supplementary material for: A Reverse Taxonomic Approach to Assess Macrofaunal Distribution Patterns in Abyssal Pacific Polymetallic Nodule Fields
Source: PLoS One. 2015 Feb 11;10(2):e0117790. doi: 10.1371/journal.pone.0117790 (PMC4324633; doi:10.1371/journal.pone.0117790)
Supplement: S4 Table — (DOCX) [file pone.0117790.s004.docx]

Electronic supplementary material to:

A reverse taxonomic approach to assess macrofaunal distribution patterns in abyssal Pacific polymetallic nodule fields

Annika Janssen^1^*, Stefanie Kaiser^1^, Karin Meißner^2^, Nils Brenke^1^, Lenaick Menot^3^, Pedro Martínez Arbízu^1^

S4 Table: Isopod MOTUs present in the French and German license area including morphological determination; la^1^= license area, g=German license area, f = French license area, seq. ident.^2^ = sequence identity,* = reference sequence.

| **clsno.** | **species ID** | **EBS#** | **exped.** | **la** | **family** | ***genus*** | ***species*** | **seq id.**  **(in %)** |
| --- | --- | --- | --- | --- | --- | --- | --- | --- |
| 0 | \| NB-Iso1 \| 06 \| BioNod´12 \| g \| Macrostylidae \| *Macrostylis* \| sp.7 sp.nov. \| 98 \| \| --- \| --- \| --- \| --- \| --- \| --- \| --- \| --- \| \| NB-Iso2 \| 06 \| BioNod´12 \| g \| Macrostylidae \| *Macrostylis* \| indet \| 98 \| \| NB-Iso3 \| 06 \| BioNod´12 \| g \| Macrostylidae \| *Macrostylis* \| sp.7 sp.nov. \| 98 \| \| NB-Iso5 \| 06 \| BioNod´12 \| g \| Macrostylidae \| *Macrostylis* \| sp.7 sp.nov. \| 98 \| \| NB-Iso343 \| 43 \| BioNod´12 \| g \| Macrostylidae \| *Macrostylis* \| sp.7 sp.nov. \| * \| \| NB-Iso344 \| 43 \| BioNod´12 \| g \| Macrostylidae \| *Macrostylis* \| indet \| 98 \| \| NB-Iso345 \| 43 \| BioNod´12 \| g \| Macrostylidae \| *Macrostylis* \| sp.7 sp.nov. \| 100 \| \| NB-Iso347 \| 43 \| BioNod´12 \| g \| Macrostylidae \| *Macrostylis* \| indet \| 99 \| | | | | | | | |
| 1 | \| NB-Iso41 \| 06 \| BioNod´12 \| g \| Haploniscidae \| *Mastigoniscus* \| sp.9 \| 99 \| \| --- \| --- \| --- \| --- \| --- \| --- \| --- \| --- \| \| NB-Iso64 \| 16 \| BioNod´12 \| g \| Haploniscidae \| *Mastigoniscus* \| sp.4 \| * \| \| NB-Iso78 \| 33 \| BioNod´12 \| g \| Haploniscidae \| *Mastigoniscus* \| sp.4. \| 99 \| \| NB-Iso79 \| 33 \| BioNod´12 \| g \| Haploniscidae \| *Mastigoniscus* \| sp.9 \| 99 \| \| NB-Iso43 \| 06 \| BioNod´12 \| g \| Haploniscidae \| *Mastigoniscus* \| sp.9 \| 99 \| | | | | | | | |
|  | NB-Iso81 | 33 | BioNod´12 | g | Haploniscidae | *Mastigoniscus* | sp.9 | 99 |
| 2 | \| NB-Iso74 \| 33 \| BioNod´12 \| g \| Macrostylidae \| *Macrostylis* \| sp.5 sp.nov. \| * \| \| --- \| --- \| --- \| --- \| --- \| --- \| --- \| --- \| \| NB-Iso75 \| 33 \| BioNod´12 \| g \| Macrostylidae \| *Macrostylis* \| sp.5 sp.nov. \| 100 \| \| NB-Iso8 \| 06 \| BioNod´12 \| g \| Macrostylidae \| *Macrostylis* \| indet \| 97 \| \| NB-Iso4 \| 06 \| BioNod´12 \| g \| Macrostylidae \| *Macrostylis* \| indet \| 97 \| \| NB-Iso76 \| 33 \| BioNod´12 \| g \| Macrostylidae \| *Macrostylis* \| sp.5 sp.nov. \| 97 \| | | | | | | | |
| 3 | \| NB-Iso192 \| 80 \| BioNod´12 \| f \| Macrostylidae \| *Macrostylis* \| sp.5 sp.nov. \| * \| \| --- \| --- \| --- \| --- \| --- \| --- \| --- \| --- \| \| NB-Iso194 \| 80 \| BioNod´12 \| f \| Macrostylidae \| *Macrostylis* \| sp.5 sp.nov. \| 98 \| \| NB-Iso195 \| 80 \| BioNod´12 \| f \| Macrostylidae \| *Macrostylis* \| sp.5 sp.nov. \| 98 \| \| NB-Iso210 \| 80 \| BioNod´12 \| f \| Macrostylidae \| *Macrostylis* \| indet \| 98 \| \| NB-Iso441 \| 101 \| BioNod´12 \| f \| Macrostylidae \| *Macrostylis* \| sp.5 sp.nov. \| 98 \| | | | | | | | |
| 4 | \| NB-Iso197 \| 80 \| BioNod´12 \| f \| Macrostylidae \| *Macrostylis* \| sp.3 sp.nov. \| * \| \| --- \| --- \| --- \| --- \| --- \| --- \| --- \| --- \| \| NB-Iso204 \| 80 \| BioNod´12 \| f \| Macrostylidae \| *Macrostylis* \| sp.5 sp.nov. \| 98 \| \| NB-Iso264 \| 73 \| BioNod´12 \| f \| Macrostylidae \| *Macrostylis* \| sp.5 sp.nov. \| 99 \| \| NB-Iso515 \| 101 \| BioNod´12 \| f \| Macrostylidae \| *Macrostylis* \| sp.5 sp.nov. \| 97 \| | | | | | | | |
| 5 | \| NB-Iso253 \| 73 \| BioNod´12 \| f \| Dendrotionidae \| *cf Dendrotion* \| aff. *thylogale* \| * \| \| --- \| --- \| --- \| --- \| --- \| --- \| --- \| --- \| \| NB-Iso256 \| 73 \| BioNod´12 \| f \| Dendrotionidae \| *cf Dendrotion* \| aff. *thylogale* \| 99 \| \| NB-Iso458 \| 101 \| BioNod´12 \| f \| Dendrotionidae \| *cf Dendrotion* \| aff. *thylogale* \| 99 \| | | | | | | | |
| 6 | \| NB-Iso245 \| 73 \| BioNod´12 \| f \| Munnopsidae \| *Acanthocope* \| *galatheae* \| * \| \| --- \| --- \| --- \| --- \| --- \| --- \| --- \| --- \| \| NB-Iso454 \| 101 \| BioNod´12 \| f \| Munnopsidae \| *Acanthocope* \| *galatheae* \| 99 \| \| NB-Iso243 \| 73 \| BioNod´12 \| f \| Munnopsidae \| *Acanthocope* \| *galatheae* \| 99 \| | | | | | | | |
| 7 | \| NB-Iso112 \| 67 \| BioNod´12 \| f \| Munnopsidae \| *Betamorpha* \| sp.1 \| * \| \| --- \| --- \| --- \| --- \| --- \| --- \| --- \| --- \| \| NB-Iso168 \| 80 \| BioNod´12 \| f \| Munnopsidae \| *Betamorpha* \| aff. *profunda* \| 100 \| \| NB-Iso170 \| 80 \| BioNod´12 \| f \| Munnopsidae \| *Betamorpha* \| aff. *profunda* \| 100 \| | | | | | | | |
| 8 | \| NB-Iso114 \| 67 \| BioNod´12 \| f \| Desmosomatidae \| *Prochelator* \| sp.2 \| 99 \| \| --- \| --- \| --- \| --- \| --- \| --- \| --- \| --- \| \| NB-Iso 230 \| 73 \| BioNod´12 \| f \| Desmosomatidae \| *Prochelator* \| sp.2 \| 100 \| \| NB-Iso 236b \| 73 \| BioNod´12 \| f \| Desmosomatidae \| *Prochelator* \| sp.2 \| * \| | | | | | | | |
| 9 | \| NB-Iso307 \| 43 \| BioNod´12 \| g \| Nannoniscidae \| *cf. Nannoniscus* \| sp. 2 \| * \| \| --- \| --- \| --- \| --- \| --- \| --- \| --- \| --- \| \| NB-Iso310 \| 43 \| BioNod´12 \| g \| Nannoniscidae \| *cf. Nannoniscus* \| sp. 2 \| 99 \| \| NB-Iso303 \| 43 \| BioNod´12 \| g \| Nannoniscidae \|  \|  \| 98 \| | | | | | | | |
| 10 | \| NB-Iso136 \| 80 \| BioNod´12 \| f \| Desmosomatidae \| *Mirabilicoxa* \| sp. 1 \| * \| \| --- \| --- \| --- \| --- \| --- \| --- \| --- \| --- \| \| NB-Iso216 \| 73 \| BioNod´12 \| f \| Desmosomatidae \| *Mirabilicoxa* \| sp. 1 \| 100 \| \| NB-Iso228 \| 73 \| BioNod´12 \| f \| Desmosomatidae \| *Mirabilicoxa* \| sp. 1 \| 99 \| | | | | | | | |
| 11 | \| NB-Iso82 \| 33 \| BioNod´12 \| g \| Desmosomatidae \| *Chelator* \| sp. 1 \| * \| \| --- \| --- \| --- \| --- \| --- \| --- \| --- \| --- \| \| NB-Iso83 \| 33 \| BioNod´12 \| g \| Desmosomatidae \| *Chelator* \| sp. 1 \| 99 \| | | | | | | | |
| 12 | \| NB-Iso95 \| 67 \| BioNod´12 \| f \| Macrostylidae \| *Macrostylis* \| sp.5 sp.nov. \| * \| \| --- \| --- \| --- \| --- \| --- \| --- \| --- \| --- \| \| NB-Iso193 \| 80 \| BioNod´12 \| f \| Macrostylidae \| *Macrostylis* \| sp.5 sp.nov. \| 99 \| | | | | | | | |
| 13 | \| NB-Iso444 \| 101 \| BioNod´12 \| f \| Munnopsidae \| *cf. Eurycope* \| *scabra* \| * \| \| --- \| --- \| --- \| --- \| --- \| --- \| --- \| --- \| \| NB-Iso460 \| 101 \| BioNod´12 \| f \| Munnopsidae \|  \| indet \| 98 \| | | | | | | | |
| 14 | \| NB-Iso353 \| 43 \| BioNod´12 \| g \| Haplomunnidae \| *Thylakogaster* \| sp.1 \| 100 \| \| --- \| --- \| --- \| --- \| --- \| --- \| --- \| --- \| \| NB-Iso354 \| 43 \| BioNod´12 \| g \| Haplomunnidae \| *Thylakogaster* \| sp.1 \| * \| | | | | | | | |
| 15 | \| NB-Iso100 \| 67 \| BioNod´12 \| f \| Haploniscidae \| *Haploniscus* \| aff. *intermedius* \| * \| \| --- \| --- \| --- \| --- \| --- \| --- \| --- \| --- \| \| NB-Iso105 \| 67 \| BioNod´12 \| f \| Haploniscidae \| *Haploniscus* \| aff. *intermedius* \| 99 \| | | | | | | | |
| 16 | \| NB-Iso104 \| 67 \| BioNod´12 \| f \| Haploniscidae \| *Haploniscus* \| aff. *intermedius* \| * \| \| --- \| --- \| --- \| --- \| --- \| --- \| --- \| --- \| \| NB-Iso268 \| 73 \| BioNod´12 \| f \| Haploniscidae \| *Haploniscus* \| aff. *intermedius* \| 100 \| | | | | | | | |
| 17 | \| NB-Iso101 \| 67 \| BioNod´12 \| f \| Haploniscidae \| *Haploniscus* \| aff. *intermedius* \| * \| \| --- \| --- \| --- \| --- \| --- \| --- \| --- \| --- \| \| NB-Iso103 \| 67 \| BioNod´12 \| f \| Haploniscidae \| *Haploniscus* \| aff. *intermedius* \| 99 \| | | | | | | | |
| 18 | \| NB-Iso42 \| 06 \| BioNod´12 \| g \| Haploniscidae \| *Mastigoniscus* \| sp.9 \| * \| \| --- \| --- \| --- \| --- \| --- \| --- \| --- \| --- \| \| NB-Iso45 \| 06 \| BioNod´12 \| g \| Haploniscidae \| *Mastigoniscus* \| sp.9 \| 99 \| | | | | | | | |
| 19 | \| NB-Iso111 \| 67 \| BioNod´12 \| f \| Munnopsidae \| *Eurycope* \| aff. *linearis* \| * \| \| --- \| --- \| --- \| --- \| --- \| --- \| --- \| --- \| \| NB-Iso276 \| 43 \| BioNod´12 \| g \| Munnopsidae \| *Eurycope* \| aff. *linearis* \| 97 \| | | | | | | | |
| 20 | \| NB-Iso241 \| 73 \| BioNod´12 \| f \| Munnopsidae \| *Eurycope* \| aff. *linearis* \| 99 \| \| --- \| --- \| --- \| --- \| --- \| --- \| --- \| --- \| \| NB-Iso497 \| 101 \| BioNod´12 \| f \| Munnopsidae \| *Eurycope* \| aff*. linearis* \| * \| | | | | | | | |
| 21 | \| NB-Iso284 \| 43 \| BioNod´12 \| g \| Munnopsidae \| *Eurycope* \| sp.6 \| * \| \| --- \| --- \| --- \| --- \| --- \| --- \| --- \| --- \| \| NB-Iso285 \| 43 \| BioNod´12 \| g \| Munnopsidae \| *Eurycope* \| sp.6 \| 99 \| | | | | | | | |
| 22 | \| NB-Iso38 \| 06 \| BioNod´12 \| g \| Desmosomatidae \| *Eugerdella* \| sp. 1 \| ^*^ \| \| --- \| --- \| --- \| --- \| --- \| --- \| --- \| --- \| \| NB-Iso56 \| 16 \| BioNod´12 \| g \| Desmosomatidae \| *Eugerdella* \| sp. 1 \| 99 \| | | | | | | | |
| 23 | \| NB-Iso226 \| 73 \| BioNod´12 \| f \| Desmosomatidae \| *Eugerdella* \| sp. 2 \| 99 \| \| --- \| --- \| --- \| --- \| --- \| --- \| --- \| --- \| \| NB-Iso236A \| 73 \| BioNod´12 \| f \| Desmosomatidae \| *Desmo. indet* \|  \| * \| | | | | | | | |
| 24 | \| NB-Iso35 \| 06 \| BioNod´12 \| g \| Desmosomatidae \| *Eugerdella* \| sp. 1 \| 99 \| \| --- \| --- \| --- \| --- \| --- \| --- \| --- \| --- \| \| NB-Iso380 \| 43 \| BioNod´12 \| g \| Desmosomatidae \| *Eugerdella* \| sp. 1 \| * \| | | | | | | | |
| 25 | \| NB-Iso36 \| 06 \| BioNod´12 \| g \| Desmosomatidae \| *Mirabilicoxa* \| sp. 2 \| * \| \| --- \| --- \| --- \| --- \| --- \| --- \| --- \| --- \| \| NB-Iso386 \| 43 \| BioNod´12 \| g \| Desmosomatidae \| *Mirabilicoxa* \| sp. 2 \| 99 \| | | | | | | | |
| 26 | \| NB-Iso33 \| 06 \| BioNod´12 \| g \| Desmosomatidae \| *Whoia* \| sp. 1 \| 99 \| \| --- \| --- \| --- \| --- \| --- \| --- \| --- \| --- \| \| NB-Iso34 \| 06 \| BioNod´12 \| g \| Desmosomatidae \| *Whoia* \| sp. 1 \| * \| | | | | | | | |
| 27 | \| NB-Iso37 \| 06 \| BioNod´12 \| g \| Desmosomatidae \| *Prochelator* \| sp. 1 \| * \| \| --- \| --- \| --- \| --- \| --- \| --- \| --- \| --- \| \| NB-Iso239 \| 73 \| BioNod´12 \| f \| Desmosomatidae \| *Prochelator* \| sp. 1 \| 97 \| | | | | | | | |
| 28 | \| NB-Iso290 \| 43 \| BioNod´12 \| g \| Nannoniscidae \| *Nannoniscus* \| sp. 1 \| * \| \| --- \| --- \| --- \| --- \| --- \| --- \| --- \| --- \| \| NB-Iso330 \| 43 \| BioNod´12 \| g \| Nannoniscidae \| *Nannoniscus* \| sp. 1 \| 99 \| | | | | | | | |
| 29 | \| NB-Iso32 \| 06 \| BioNod´12 \| g \| Isopoda indet \|  \| sp.1 \| * \| \| --- \| --- \| --- \| --- \| --- \| --- \| --- \| --- \| \| NB-Iso89 \| 33 \| BioNod´12 \| g \| Isopoda indet \|  \| sp.1 \| 99 \| | | | | | | | |
